# Quantitative estimates of the regulatory influence of long non-coding RNAs on global gene expression variation using TCGA breast cancer transcriptomic data

**DOI:** 10.1371/journal.pcbi.1012103

**Published:** 2024-06-05

**Authors:** Xiaoman Xie, Saurabh Sinha

**Affiliations:** 1 Center for Biophysics and Quantitative Biology, University of Illinois Urbana-Champaign, Urbana, Illinois, United States of America; 2 Wallace H. Coulter Department of Biomedical Engineering at Georgia Tech and Emory University, Georgia Institute of Technology, Atlanta, Georgia, United States of America; 3 H. Milton Stewart School of Industrial and Systems Engineering, Georgia Institute of Technology, Atlanta, Georgia, United States of America; Penn State University: The Pennsylvania State University, UNITED STATES

## Abstract

Long non-coding RNAs (lncRNAs) have received attention in recent years for their regulatory roles in diverse biological contexts including cancer, yet large gaps remain in our understanding of their mechanisms and global maps of their targets. In this work, we investigated a basic unanswered question of lncRNA systems biology: to what extent can gene expression variation across individuals be attributed to lncRNA-driven regulation? To answer this, we analyzed RNA-seq data from a cohort of breast cancer patients, explaining each gene’s expression variation using a small set of automatically selected lncRNA regulators. A key aspect of this analysis is that it accounts for confounding effects of transcription factors (TFs) as common regulators of a lncRNA-mRNA pair, to enrich the explained gene expression for lncRNA-mediated regulation. We found that for 16% of analyzed genes, lncRNAs can explain more than 20% of expression variation. We observed 25–50% of the putative regulator lncRNAs to be in ‘cis’ to, i.e., overlapping or located proximally to the target gene. This led us to quantify the global regulatory impact of such cis-located lncRNAs, which was found to be substantially greater than that of trans-located lncRNAs. Additionally, by including statistical interaction terms involving lncRNA-protein pairs as predictors in our regression models, we identified cases where a lncRNA’s regulatory effect depends on the presence of a TF or RNA-binding protein. Finally, we created a high-confidence lncRNA-gene regulatory network whose edges are supported by co-expression as well as a plausible mechanism such as cis-action, protein scaffolding or competing endogenous RNAs. Our work is a first attempt to quantify the extent of gene expression control exerted globally by lncRNAs, especially those located proximally to their regulatory targets, in a specific biological (breast cancer) context. It also marks a first step towards systematic reconstruction of lncRNA regulatory networks, going beyond the current paradigm of co-expression networks, and motivates future analyses assessing the generalizability of our findings to additional biological contexts.

## Author summary

Many studies have reported the role of long non-coding RNAs in regulating gene expression, yet there has not been a systematic assessment of the extent to which gene expression variance can be explained by lncRNA regulation. lncRNA-mRNA co-expression networks are commonly constructed using pair-wise correlations, but these approaches do not claim to quantify lncRNA-driven regulation and may be confounded by shared transcriptional regulators of lncRNA-mRNA pairs. Here, we used a multivariable linear regression model to predict mRNA expression using lncRNAs’ expression in a large breast cancer transcriptomic dataset from TCGA, and obtained estimates for the overall gene expression variance under lncRNA regulation. We also integrated orthogonal criteria such as genome proximity and protein binding information to refine these estimates and identified lncRNA-mRNA associations that are both statistically significant and biologically supported.

## Introduction

Long non-coding RNAs (lncRNA) are a class of RNA transcripts that are longer than 200bp and not translated to ORFs longer than 100 amino acids [1]. Over 10,000 lncRNAs have been annotated in the human genome, yet their functional characterization lags far behind that of protein-coding genes. There have been speculations that most lncRNAs are by-products of leaky transcription and other cellular processes, without important functions [2,3]. At the same time, there are many studies demonstrating evidence of lncRNA function and it is generally believed that such functions are carried out via their regulatory influence on other genes [4–7]. Accordingly, numerous attempts at determining co-expression networks involving lncRNAs and protein-coding genes have been published [8–12]. Despite these efforts, a simple but fundamental question remains unanswered regarding lncRNA-driven regulation: to what extent can gene expression variation across individuals be attributed to lncRNA-driven regulation? Our work attempts to answer this key question by systematic statistical analysis of a large transcriptomic data set and reconstruction of a conservative lncRNA regulatory network, where putative lncRNA-mRNA relationships are inferred while the regulation of TFs and other lncRNAs are controlled and additional criteria, such as genomic proximity and protein-dependent co-expression are imposed.

lncRNAs as a class have received special attention in the context of cancer. Several lncRNAs have been found to have critical roles in cell proliferation, invasion and apoptosis [13,14] [15–18]. The Cancer LncRNA Census, as part of the ICGC/TCGA Pan-Cancer Analysis, reported 122 lncRNAs to have causal function in cancer phenotypes [19]. Some of the well-studied lncRNAs such as MALAT1 and NEAT1 are known for their causal impact on breast cancer metastasis [20–23]. Given their emerging significance in breast cancer, we conducted our systematic characterization of the extent of lncRNA regulatory influence using transcriptomic data from a cohort of breast cancer patients in the Cancer Genome Atlas (TCGA). We hoped that this may lead to a useful catalog of important lncRNA regulators of this disease, in addition to providing broadly applicable insights into the central question raised above.

A quantitative assessment of lncRNA regulation can be performed by examining co-expression patterns in cohort-level transcriptomic data and determining putative lncRNA regulators of protein-coding genes. Previous studies constructed co-expression networks between lncRNAs and protein coding genes using methods such as weighted correlation network analysis (WGCNA) [5,24], examining correlations between the expression levels of each lncRNA-mRNA pair [25]. However, co-expression between a lncRNA and a mRNA does not imply that the lncRNA regulates the gene. One possible reason is that both the lncRNA and the mRNA are regulated by the same transcription factor (TF). Being co-regulated by the same TF regulators may cause high correlation between the expression levels between two transcripts, e.g., a lncRNA and an mRNA. Thus, accounting for evidence of relationships with TFs when detecting lncRNA-mRNA co-expression is expected to enrich for regulatory interactions between lncRNAs and mRNAs. Another important consideration in building regulatory networks from expression analysis is the possibility of multiple regulators influencing a target gene; indeed, this is the common practice in building TF-gene regulatory networks, where multi-variable regression/classification models are used to explain a target gene’s expression as a function of multiple TFs [26–29]. A co-expression network that examines each lncRNA-mRNA pair independently for evidence of correlated expression ignores this facet of regulation, and is likely to have false as well as missing edges as a result. Finally, a methodological challenge that arises when building lncRNA-mRNA regulatory networks is that there are a large number of lncRNAs to select the true regulators from and this poses statistical difficulties, especially due to extensive co-expression among lncRNAs themselves. A possible solution is to demand that a single mRNA’s expression is explained using only a small set of regulators, chosen from the full complement of lncRNAs in a data-driven manner.

We sought to address the above shortcomings of co-expression analysis in determining lncRNA-mRNA regulatory relationships and estimating the fraction of expression variation in TCGA breast cancer samples that can be explained using lncRNAs as regulators. Notably, a diversity of mechanisms has been reported for lncRNA-driven regulation, including interference in transcription by lncRNA genes overlapping the target gene [30], cis-regulatory action by lncRNAs located proximally to a gene [31,32], trans-effects such as competing endogenous RNA (ceRNA) [33], etc. [34] We sought to objectively estimate the extent to which each mechanism is borne out in the TCGA breast cancer data, adopting a simple strategy: modeling each target gene’s expression while limiting the candidate lncRNA regulators to those for which there is plausible evidence of a particular mechanism being at play. Moreover, there are reports in the literature that the regulatory influence of lncRNAs may in some cases depend on the presence of proteins such as the DNA binding TFs or RNA-binding proteins (RBPs), e.g., if the lncRNA acts as a scaffold for assembly of complexes involving such proteins along with DNA and/or RNA. We hypothesized that such a protein-dependent regulatory relationship between a lncRNA and its target genes may leave its footprint in the expression data, by way of “interaction terms” in a multi-variate regression of gene expression against lncRNA and TF/RBP expression. We therefore sought to detect such examples of protein-dependent lncRNA action via statistical testing of interaction terms.

With the above goals in mind, we analyzed the transcriptome data for 13,963 protein coding genes and 1,079 lncRNAs of 1,217 breast cancer tumor samples from TCGA. We assessed how much variance of the gene expression can be explained by lncRNAs using linear regression models that treat expression values of selected lncRNAs as independent variables (“features”). Importantly, we accounted for potential confounding effects of TFs as common regulators of a co-expressed lncRNA-mRNA pair in performing the regression analysis. We repeated the analysis using lncRNAs that can be potentially associated with the target gene under a specific mechanistic assumption, such as lncRNAs that overlap the gene, or lncRNAs that are located in the same Topologically Associating Domain (TAD) as the target gene, or lncRNAs that may compete with the target gene for the same microRNA, i.e., the ceRNA mechanism. In any modeling step where a large number of candidate regulators were possible, we controlled for model complexity by adopting the Elastic Net approach [35] that ensures sparsity of selected features, and by assessing the percentage of expression variance explained in an unseen part of the data set. Statistically significant interactions between lncRNA and RBPs/TFs were detected by first training models to explain the target gene expression using both lncRNAs and the class of proteins, and then assessing the added value of including interaction terms in the model.

Combining the results of the above-mentioned analyses, we finally created a lncRNA-mRNA regulatory network with edges being lncRNA-mRNA connections that are strongly supported by the above models and by additional evidence of a plausible regulatory mechanism. About 25%-50% of the predicted edges are in ‘cis’ to, i.e., overlapping or located proximally to the target gene. We found that lncRNAs with prominent roles in the reconstructed network are supported by literature-based evidence to have important regulatory roles in the context of breast cancer. We also made the intriguing observation that genes targeted by specific lncRNAs, according to the network, are highly enriched for functions related to protein translation. In summary, this work presents a comprehensive and rigorous analysis of expression relationships involving lncRNAs and protein coding genes, along with TFs and RBPs, borne out by TCGA breast cancer data. It also presents a blueprint for identification of significant regulatory lncRNAs and their regulons in any data set of individual-level expression variation.

## Results

### Breast cancer transcriptomes reveal extensive co-expression involving lncRNAs

**Fig 1:**
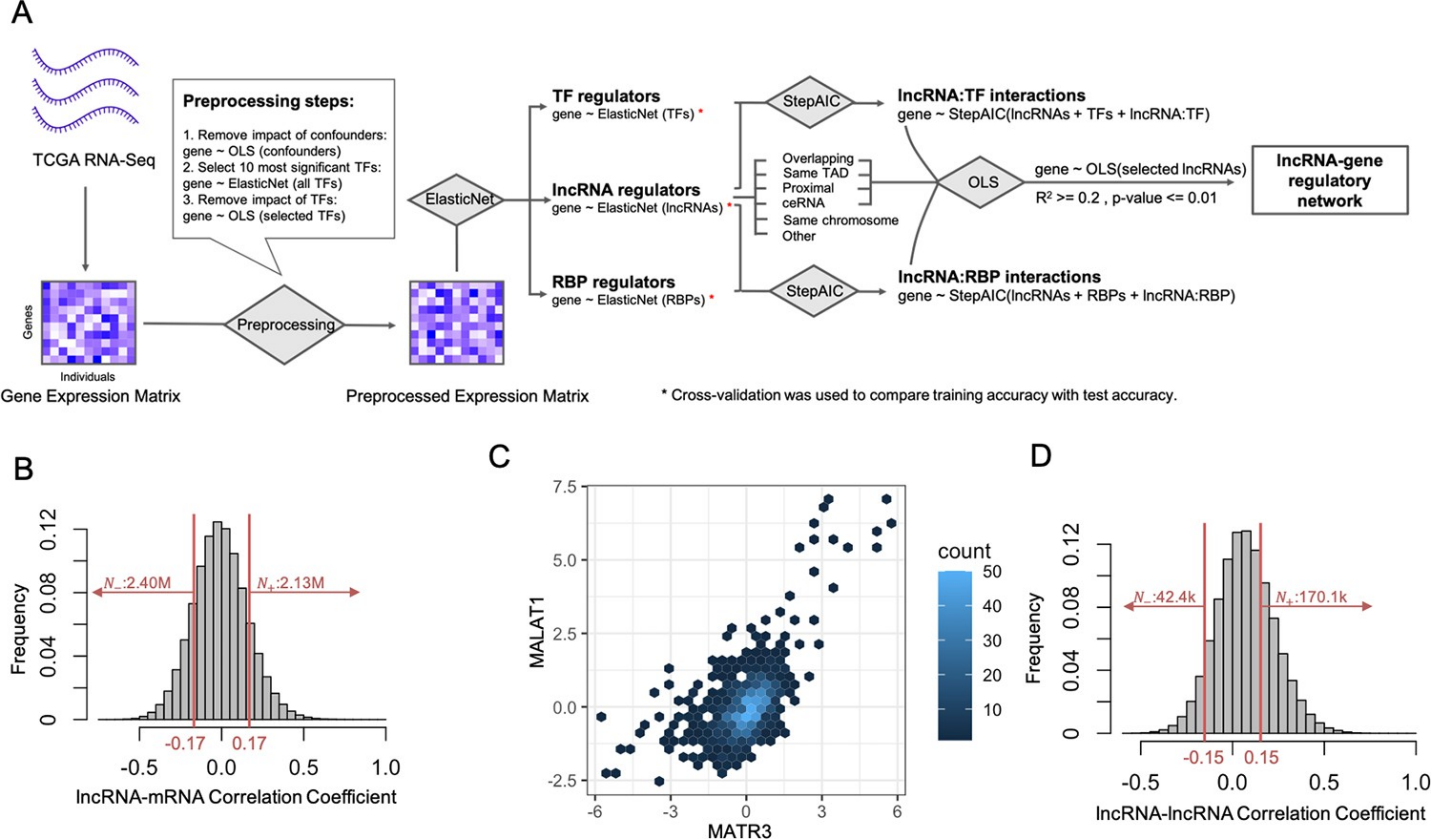
Co-expression among mRNAs and lncRNAs. (A) Graphical Abstract of Workflow. RNA-Seq data for 1,217 primary tumor samples from 1,092 breast cancer patients were obtained from TCGA-BRCA cohort. The processed transcriptomic data (see Methods) include expression profiles for 1,079 lncRNAs and 13,963 mRNAs. Using ElasticNet regression, significant lncRNA, TF, and RBP regulators were identified. These significant lncRNAs with their supporting mechanisms or involved in interaction terms were then used in an Ordinary Least Squares (OLS) regression model to construct the final lncRNA-regulatory network. (B) Histogram of Pearson correlation coefficients of lncRNA-mRNA pairs. Among ~15M pairs analyzed, ~4.5M pairs had absolute value of Pearson correlation above 0.17, which corresponds to Bonferroni corrected p-value of 0.05. (C) Density scatter plot of expression of lncRNA MALAT1 and mRNA MATR3. MALAT1 and MATR3 are highly correlated with Pearson correlation of 0.58 (Bonferroni corrected p-value of 1.45e-109). (D) Histogram of pairwise Pearson correlations between lncRNA-lncRNA pairs. There are ~170K pairs positively correlated with correlation greater than 0.15 and 42K negatively correlated pairs with correlation lower than -0.15 (Bonferroni corrected p-value of 0.05).

We analyzed the transcriptomes of 1,217 primary tumor samples from breast cancer patients (source: TCGA), with the goal of identifying regulatory relationships between lncRNAs and mRNAs. TCGA HTSeq-counts data was preprocessed to remove lowly expressed genes, normalize expression values and control for common confounding variables, such as age, sex, ethnicity and race. (See Methods for details of data processing and normalization.) The processed data included expression values for 13,963 mRNAs and 1,079 lncRNAs (**Fig 1A**). In a preliminary analysis, we calculated Pearson correlation coefficients (PCC) for each lncRNA-mRNA pair, and found extensive co-expression (**Fig 1B**), with 30% of all pairs at a PCC of absolute value greater than 0.17 (Bonferroni corrected p-value of 0.05). An example of a pair with strong correlation is the lncRNA MALAT1, a key regulator in breast cancer [22], and the mRNA MATR3, an RNA-binding protein with tumor suppressive function in breast cancer [36] (**Fig 1C**). The extensive co-expression was also noted among pairs of lncRNAs (**Fig 1D**), with ~36.5% of all pairs at a PCC of absolute value greater than 0.15 (Bonferroni corrected p-value of 0.05), with the majority of these co-expressed pairs being positively correlated. The extensive co-expression observed among lncRNAs and mRNAs is consistent with previous analyses of cancer data sets [8,9,11], including the TCGA breast cancer cohort [12,37], and provides a natural starting point for the rest of this work.

### lncRNAs can explain a significant portion of gene expression variation

**Fig 2:**
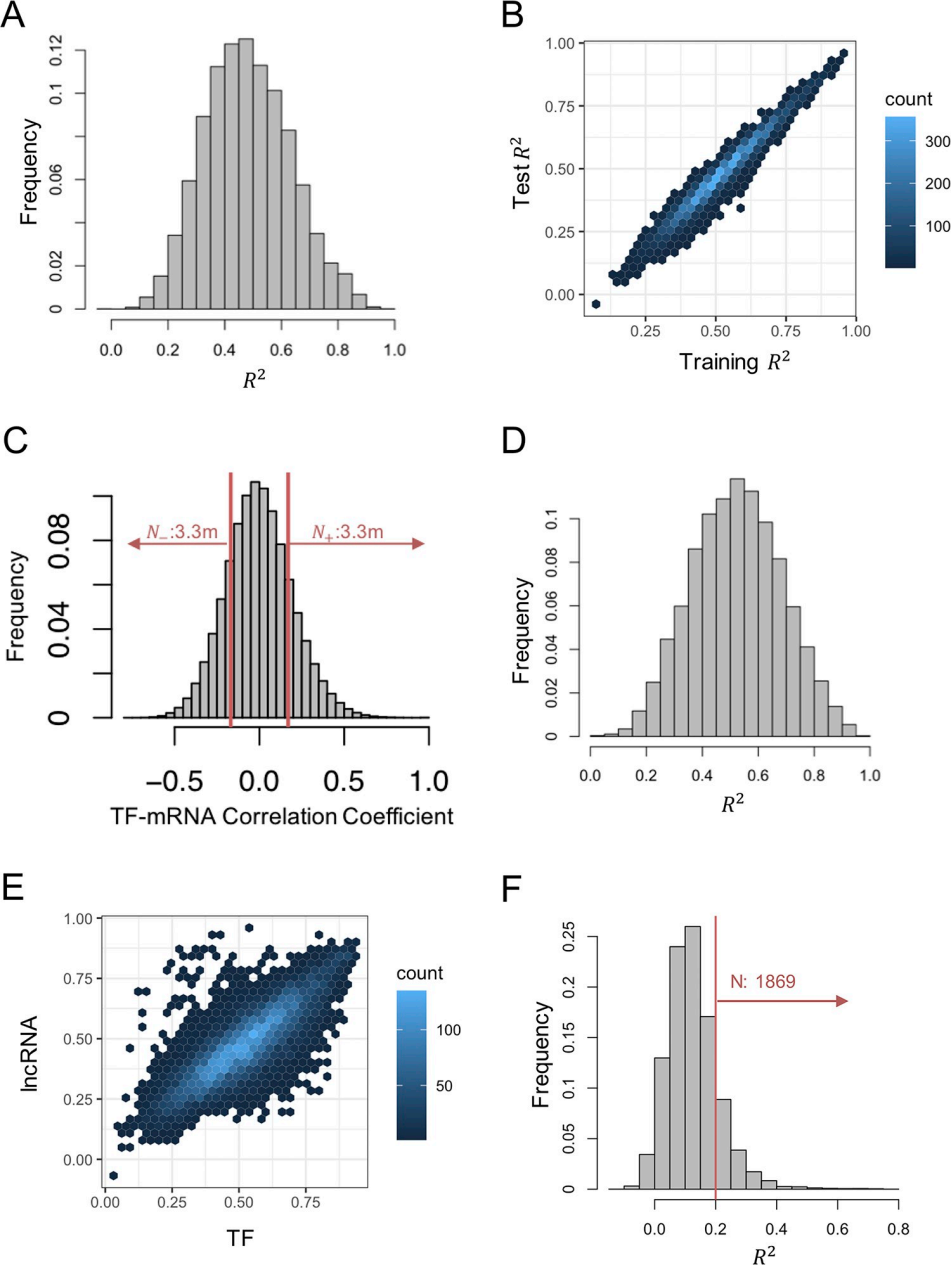
LncRNA-mRNA co-expression network. (A) Histogram of test *R*^2^ when mRNA expression values were modeled using lncRNA. For each mRNA, Elastic Net algorithm was used to select ~10 most significant lncRNAs as predictors, which were then used to predict the mRNA’s expression using ordinary least squares (OLS) regression. Average test *R*^2^ in a 5-fold cross-validation was calculated for each mRNA. (Each “fold” involved selecting predictor lncRNAs and training the model on 80% of samples and testing on the remaining 20%.) (B) Density scatter plot of test *R*^2^ and overall (“training”) *R*^2^ for the models. Overall *R*^2^ is calculated by training the model on all samples and calculating its *R*^2^ on all samples. Average test *R*^2^ values were generally similar to the corresponding overall *R*^2^ values of the same gene, indicating that overfitting is not a major concern. (C) Histogram of pairwise Pearson correlations for TF-mRNA pairs. About 6.6M of the pairs (37.57% of all pairs) have a PCC with absolute value greater than 0.17 (Bonferroni corrected p-value of 0.05). (D) Histogram of test *R*^2^ when mRNA expression values were modeled using TFs selected using Elastic Net, in a five-fold cross validation. (E) Scatter plot of test *R*^2^ values for models utilizing TFs or lncRNAs (x and y axes respectively) as predictors. Each point represents an mRNA whose expression is predicted by the models. PCC between two axes is 0.82 (p-value < 2.2e-16). (F) Histogram of test *R*^2^ values (average over five folds of cross-validation) when lncRNAs were used to model mRNA expression values from which TF-based predictions have been subtracted away.

Our next goal was to estimate how much of the variation in gene expression can be explained using lncRNAs. This is an important step towards quantifying the potential regulatory influence of lncRNAs as a class. To this end, we first trained a linear model to predict each gene’s expression as a weighted sum of multiple lncRNAs’ expression levels. With over 1000 lncRNAs being candidate predictors and ~1,200 samples available for modeling, it was imperative that we constrain the model’s complexity and avoid “over-fitting” the data. We therefore used the Elastic Net algorithm to select ~10 informative lncRNAs for each target gene (mRNA) and used only the selected short-list of lncRNAs in modeling that gene (see Methods). The median R^2^ (percentage of variance explained) was noted at 0.52 (**Fig A in S1 Text**), meaning that for most genes over half of the variance in expression can be explained using ~10 selected lncRNAs. To ensure that this observation is not the result of over-fitting, we performed a 5-fold cross-validation of the model. The test R^2^ values (**Fig 2A**) had a median of 0.47, which is close to the 0.52 value noted above. **Fig 2B** shows that the test R^2^ value of a gene is generally close to the overall R^2^ value observed when training and evaluating the linear model on all samples, indicating that the results are likely not the result of over-fitting. The high R^2^ values noted here are also mirrored in high values of Pearson Correlation Coefficient (PCC) between predicted and observed expression levels (**Figs B and C in S1 Text)**, with a median PCC of 0.66.

The observation that a substantial portion of the variance of mRNA expression can be explained using small numbers of lncRNAs suggests extensive regulation by lncRNAs. But such lncRNA-mRNA covariation also reflects the effects of shared regulators such as transcription factors (TFs) that influence the expression of both the mRNA and the lncRNA. To arrive at a more conservative estimate of the extent of influence and to enrich the statistically identified lncRNA-mRNA pairs for those with direct regulatory relationships (lncRNA regulates mRNA), we considered two options: (a) impose mechanistic prior knowledge, as is done by demanding the presence of TF binding sites in gene promoters when reconstructing TF-gene regulatory networks [38], and/or (b) remove the effect of other regulatory mechanisms, e.g., TF-driven regulation of a lncRNA-mRNA pair, by treating them as statistical confounders.

We first chose to adopt the latter approach of controlling for shared regulators (TFs). A correlation analysis (**Fig 2C**) revealed that ~6.6M TF-mRNA pairs (37.6% of all pairs) have a PCC with absolute value greater than 0.17 (Bonferroni corrected p-value of 0.05), highlighting their extensive co-expression as expected. We then trained linear models to predict mRNA expression using ~10 selected TFs (from all 1,255 TFs included in the data set) and evaluated these using 5-fold cross validation. Test R^2^ values, shown for all target genes in **Fig 2D**, have a median of 0.52, which is somewhat higher than the median R^2^ value of 0.47 in comparable evaluations of lncRNA-based predictions. Direct comparison of R^2^ values from TF-based and lncRNA-based models (**Fig 2E**) suggests that genes tend to be predictable to similar degrees using either class of predictors (TFs or lncRNAs). We also observed genes that are far more predictable using lncRNAs only or using TFs only (off-diagonal points in **Fig 2E**). Focusing on the 109 mRNAs that are more predictable using lncRNAs (y–x > 0.2 and y > 0.5 in **Fig 2E**) revealed that the most common significant lncRNA predictors of these target genes are AC108449.2 and AC073896.4 (**S1 and S2 Tables**), increasing our confidence in their functional regulatory roles. For example, AC073896.4 is sense overlapping to gene SMARCC2, which is one of the core subunits of human SWI/SNF complex, which is known to be associated with breast cancer [39]. Examining the most significant (p-value < 1E-10) lncRNA-mRNA relationships involving the above set of 109 mRNAs, we observed that 23.7% (65/274) were overlapping pairs and 39.8% (109/274) were pairs with genes within 1 Mbp of each other. These observations are consistent with reported mechanisms of lncRNA action that involve gene overlap [40,41], including in breast cancer context [42], as well as proximal chromatin localization [43]. We visit these observations again in a later section.

Finally, for each mRNA we computed the residuals from its TF-based model, i.e., subtracted from each original expression value the prediction from the TF-based model, and trained lncRNA-based models on these residuals. In other words, we now sought to use lncRNAs to explain the expression variance remaining after the putative effects of TFs had been subtracted away. **Fig 2F** shows the distribution of test R^2^ values for all genes. (Also see **Fig D in S1 Text** for overall R^2^ values.) Clearly, these estimates of percentage variance explained are substantially lower than those from **Fig 2A** (before TF effects were subtracted), with a median R^2^ of 0.12 compared to 0.47, which is consistent with our expectation that much of the lncRNA-mRNA covariation can be explained as the result of TF-driven regulation. At the same time, we noted that the R^2^ value was 0.2 or more for 1,869 mRNAs (16.2% of all); i.e., for a substantial number of mRNAs, at least 20% of the variance may be the result of lncRNA-driven regulation. While this threshold of 20% is somewhat arbitrary, it does represent a highly significant level of statistical association: a corresponding null distribution estimated empirically by permuting the expression levels of target genes across samples revealed that none of the 11,531 target genes can be modeled at a level better than this threshold (**Fig E in S1 Text**).

The high R^2^ values observed above for a subset of target genes might still reflect mostly their co-expression with lncRNAs as opposed to regulatory influence of those lncRNAs, due to unknown shared regulators whose effects could not be subtracted away. We thus performed a “control” exercise where we used a random set of 1,079 enzyme-encoding mRNAs in place of the 1,079 lncRNAs as predictors and obtained R^2^ values for all genes in an analogous manner. Since enzyme-encoding mRNAs do not represent a regulatory class, prediction of a target gene’s expression using these mRNAs likely reflects co-expression rather than regulation. Repeating this control exercise 100 times, each time with a different random choice of 1,079 enzyme-encoding mRNAs as predictors, we obtained a null distribution of R^2^ values for each target gene; this allowed us to assign empirical p-values to the R^2^ value achieved for each target gene when using lncRNAs as predictors. We observed 699 genes to have empirical p-value < = 0.01 (578 genes have p-value < 0.01, **S3 Table**), i.e., where at most one of the 100 control exercises using enzyme-encoding mRNAs achieved an R^2^ value better than that seen with lncRNA predictors. These 699 genes comprise 6.1% of the 11,531 target genes analyzed, significantly higher than the chance expectation of 1% (Binomial test p-value <1.11e-302). This suggests that the high R^2^ values observed using lncRNA predictors do not merely represent co-expression and are likely to reflect lncRNA-related regulation.

In summary, our analyses provide a rough estimate of the part of transcriptomic variation that may be under lncRNA regulation, and points to the existence of a large number of genes under such regulation, even after accounting for the confounding effects of common regulators.

### Categorization of putative lncRNA regulators based on location relative to target gene

**Fig 3:**
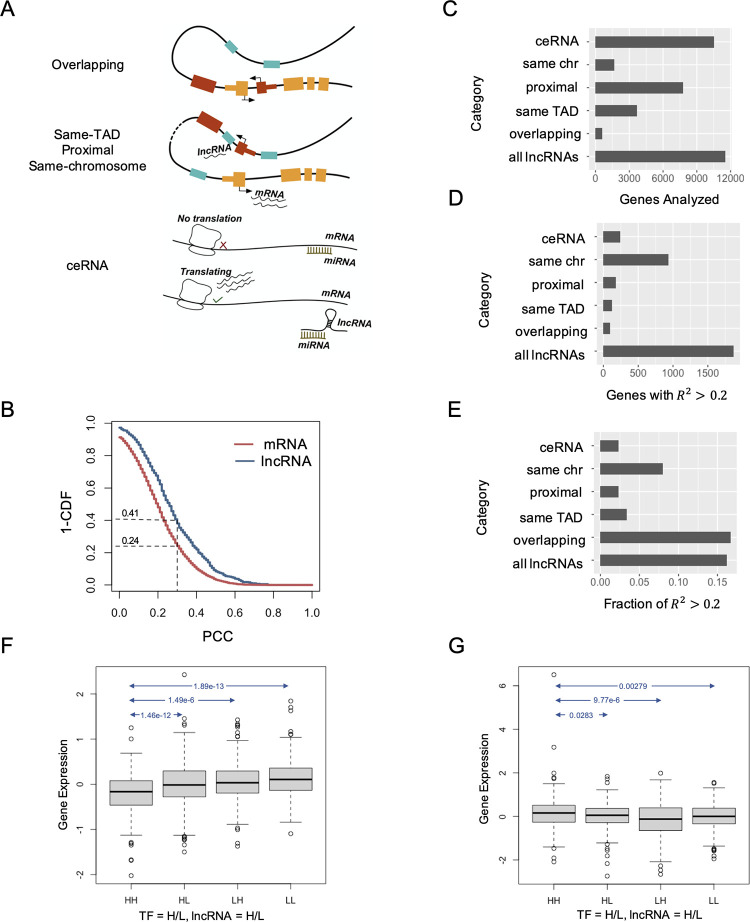
Contribution of lncRNAs from different “mechanistic” categories. (A) Schematic of lncRNA regulation mechanisms. The figure shows the four classes of lncRNAs acting in cis with the target gene and one type of trans-acting lncRNAs (ceRNA). (B) Overlapping lncRNA-mRNA pairs (blue) are more correlated than overlapping pairs of mRNAs (red). X-axis shows the Pearson Correlation Coefficient (PCC) and the Y-axis shows for any value of PCC what fraction of lncRNA-gene pairs have correlation above that value. (CDF refers to cumulative distribution function.) (C) Number of genes whose expression was modeled using lncRNAs from each category. For instance, only 588 genes had an overlapping lncRNA to use as predictor, thus only these genes could be modeled. (D,E) Number of genes (D) and fraction of genes (E) that were modeled well (test *R*^2^ > 0.2, on average across five folds of cross-validation) using lncRNAs from different categories. The fraction (E) is calculated as the ratio of respective numbers from C and D. (F) Normalized expression of gene LRP1 in four groups of samples where lncRNA TMPO-AS1 and the TF EBF1 are expressed at above (high) or below (low) their respective median level. In each category label, the first ‘H/L’ indicates high/low expression of TF and the second ‘H/L’ corresponds to the lncRNA. LRP1 expression is significantly lower in samples where both the TF and the lncRNA are highly expressed (HH) compared with the other three groups where either the TF or the lncRNA or both are lowly expressed (t-test p-values are shown for each between group comparison). The interaction term representing EBF1 and TMPO-AS1 has a p-value of 3.23E-10 in modeling gene LRP1. (G) Gene MYL12A expression is significantly higher in samples with high levels of RBP FXR1 and lncRNA OIP5-AS1 (HH) compared to the three other groups (HL, LH or LL) where either regulator has low expression. The interaction term representing FXR1 and OIP5-AS1 has a p-value of 7.8E-17 in modeling gene MYL12A.

We next sought to characterize putative lncRNA regulators identified by our models above in terms of potential mechanisms of action. There have been several reports of cis-regulatory effects of lncRNAs (**Fig 3A)**, e.g., antisense lncRNAs that regulate neighboring genes pre-transcriptionally, transcriptionally or post-transcriptionally [30]. Indeed, global maps of lncRNA-DNA interaction show that nucleus-retained lncRNAs tend to localize on proximal chromatin regions [43]. On the other hand, an often-reported mechanism of action is the so-called competing endogenous RNA (ceRNA) [44], which manifests in trans. It refers to the situation where a microRNA regulates a target mRNA post-transcriptionally and a lncRNA capable of binding the microRNA competes for the latter, thereby indirectly regulating the mRNA (**Fig 3A**). To assess the relative frequencies of cis and trans regulatory mechanisms, we considered, for each target gene, the following categories of associated lncRNAs: those (A) sense or antisense overlapping with target gene (“overlapping”), (B) located within the same topologically association domain (TAD) detected from Hi-C data from MCF10A breast cancer cell lines [45]; [46] as the target gene (“same-TAD”), (C) located within 1 Mbp of the target gene (“proximal”), (D) located on the same chromosome as the target gene (“same-chromosome”) and (E) competing endogenous RNAs (“ceRNAs”) predicted to share a microRNA regulator with the target gene, based on microRNA-mRNA and microRNA-lncRNA interactions [47]. In our context, competing endogenous RNA refers to a lncRNA-mRNA pair that are targets of the same miRNA in the same cellular contexts (see Methods). We focused on the target mRNAs whose expression could be well explained using lncRNA regulators (R^2^ > = 0.2 and empirical p-value < 0.01, **S3 Table**), and examined their strongest putative lncRNA regulators (p-value < = 1E-10 in OLS regression, and pair-wise correlation greater than 0.1 in absolute value) in terms of the above categories. This yielded 643 lncRNA-mRNA associations (**S4 Table**), of which 500 (77.8%) were positive and 143 (22.2%) were negative relationships. Of these, 171 pairs have correlation lower than 0.2 and thus might not be detected by traditional co-expression based methods.

We noted that 177 (27.5%) of these pairs were overlapping lncRNA-mRNA pairs, all but one of which were positively correlated. Similarly, 228 (35.5%) of the 643 pairs were within the same TAD as each other; 54.0% and 62.7% were proximal and on the same chromosome (respectively) as each other, almost always positively correlated (**Table 1**). In other words, the discovered associations had large fractions of pairs located in “cis” and such pairs were overwhelmingly found to be positively related pairs. We also confirmed that these large fractions are not expected merely by chance (**S2 Text**). We noted a more balanced ratio of positively correlated pairs (51.5%) and negatively correlated pairs (48.5%) for the 293 trans lncRNA-mRNA pairs.

**Table 1:** lncRNA-mRNA pairs categorized by relative genomic locations. For mRNAs that could be modeled using lncRNAs with R^2^ > = 0.2 and empirical p-value < 0.01, we considered putative lncRNA regulators with OLS p-value < = 1E-10 and pairwise correlation coefficient > = 0.1 in absolute value. Shown are the number of pairs in each category and the number (of those) that are positively correlated. “Non-proximal” refers to pairs on different chromosomes or more than 1 Mbp apart on the same chromosome.

	Number of Annotated Pairs (out of 643 pairs)	Positively Correlated
Overlapping	177 (27.5%)	176
Same TAD	228 (35.4%)	228
Proximal	347 (54.0%)	346
Same Chromosome	403 (62.7%)	394
ceRNA	41 (6.4%)	31
Non-proximal	296 (46.0%)	154

A previous study by Chen et al. [48] has reported greater expression correlation between overlapping protein-coding genes than between non-overlapping pairs of genes. We therefore asked if the prevalence of overlapping pairs (and by generalization, the cis-located pairs) noted above may simply reflect co-expression rather than regulator-target relationships. To answer this, we considered 3,715 pairs of overlapping protein-coding genes (PCGs) and computed pairwise correlations to obtain a null distribution, which we then contrasted with a corresponding distribution of 615 overlapping lncRNA-mRNA pairs selected in an unbiased manner, without regard to their expression profiles. As shown in **Fig 3B**, the two distributions are significantly different (Kolmogorov-Smirnoff test p-value 2.1e-14). For instance, 250 (41%) of the 615 overlapping lncRNA-mRNA pairs have a correlation coefficient > = 0.3, a ~1.6 fold enrichment over the corresponding fraction of 24% seen among overlapping PCG pairs (Binomial test p-value of 1.8e-19). In other words, a random overlapping lncRNA-mRNA pair has a significantly higher expression correlation than a random overlapping PCG pair, making such overlaps a marker of potential regulatory relationship. This also suggests that the 177 overlapping lncRNA-mRNA pairs identified by us (**Tables 1 and S4**) are enriched for regulatory pairs.

Taken together, our statistical analyses suggest various modes of cis-regulatory action as a prominent theme in lncRNA-driven regulation. We also noted 288 lncRNA-mRNA pairs (of the 643 in **S4 Table**) whose genes were located more than 1 Mbp away on the same chromosome or on different chromosomes. These were evenly split between positive and negative correlations (146 and 142 respectively), in sharp contrast to cis-located pairs, which were predominantly positively correlated. (See **S3 Text** for selected examples of such pairs that have literature support for their regulatory relationship.) Overall, of the 288 significant lncRNA-mRNA pairs that were located distally or in trans, only 19 were designated as ceRNA in our analysis, leaving the vast majority of such pairs mechanistically unresolved.

### cis-located lncRNAs explain a substantial part of gene expression variation

Above, we observed a prevalence of cis location in a lncRNA regulatory network that was obtained without regard to lncRNA location or mechanism. We next sought to quantify the percentage of mRNA expression variation that can be explained by lncRNA regulators limited to each of the above-defined categories of cis action. To this end, we repeated the above modeling exercise (**Fig 2F**) while limiting the candidate set of lncRNAs for a target gene to each of the five categories–overlapping, same-TAD, proximal, same-chromosome or ceRNA. The newly imposed restrictions on candidate lncRNAs used for predicting a given gene’s expression led to reductions in the number of genes analyzed (**Fig 3C**). We next examined the number (**Fig 3C**) and fraction (**Fig 3D**) of target genes that could be modeled “well” (average test R^2^ > = 0.2 in 5-fold cross validation) using each of the five categories of candidate lncRNAs. The “overlapping” category yielded the highest fraction, with ~16.7% of the 588 genes modeled using this class of lncRNAs reaching the R^2^ > = 0.2 threshold. This fraction is similar to the fraction of genes modeled well (16.2%) when the unrestricted class of all 1079 lncRNAs is used as candidates. Among the 588 genes that do have at least one overlapping lncRNA, 303 genes can be modeled well using all lncRNAs as candidates and 32% of these can also be modeled well using just the overlapping lncRNA(s) (**Fig F in S1 Text**). In other words, if an mRNA’s expression can be predicted using lncRNAs and the mRNA has an overlapping lncRNA, then that overlapping lncRNA is likely to be a significant predictor. (Also see **Fig G in S1 Text**) Note that it is possible for such pairs to be co-regulated by a shared regulator, rather than the lncRNA regulating the mRNA, but our strategy of subtracting the predicted effects of TFs prior to modeling makes this less likely. These results reinforce the observations made above and in the literature [49] that lncRNA-gene overlaps are a significant marker of and potentially tied to the underlying mechanisms of lncRNA regulatory function.

When modeling a gene using candidate lncRNAs in the “same-TAD” (typically, 1.8 candidates per gene) or “proximal” (typically, 2.0 candidates per gene) categories, the fraction of genes with R^2^ > = 0.2 fell to 2–4% (**Fig 3E**), much smaller than the 16.7% observed with “overlapping” lncRNAs. This is not surprising: a TAD often has multiple genes and it is possible that a lncRNA within the same TAD regulates only one of those genes, driving the above-mentioned fraction down. Indeed, we found that ~20% of lncRNA-harboring TADs include at least one gene that can be modeled well using lncRNAs in the same TAD. In contrast, among TADs harboring enzyme-encoding genes, only 8.8% include at least one gene that can be modeled well using enzymes in the same TAD.

Enlarging the candidate sets even further, we noted that ~8.0% of analyzed genes can be predicted with R^2^ > = 0.2 using lncRNAs from the same chromosome (typically, using ~11.5 regulators per gene). One way to interpret this observation is that while ~1,900 (~16%, **Fig 2F**) of analyzed genes could be modeled well using cis- as well as trans-acting lncRNAs, about 50% of these are amenable to expression prediction using only (putatively) cis-acting lncRNAs from the same chromosome.

Finally, gene expression prediction using lncRNAs in the ceRNA category led to only ~2% (246 out of 10556) of analyzed genes being modeled well, even though an average of 11 lncRNAs were considered as candidate regulators for each gene (**S6 Table**). We found 45 genes that have R^2^ equal to 0.2 or greater and empirical p-value < 0.01 (**S4 Text**) in such modeling. Notable among these, lncRNA GAS5 was selected as one of the significant predictors for 18 out of the 45 mRNAs (**Table 2**), including 12 genes that encode ribosomal proteins, including PRS18, RPL31, RPL30, etc.. A previous study found that the overexpression of GAS5 facilitates cell apoptosis in breast cancer and increases the sensitivity to treatments [50], and it has been shown to act via the ceRNA mechanism [51]. It belongs to the 5’-TOP gene family that includes all ribosomal proteins [50], and five of its twelve putative ribosomal protein targets were also found to be among the top 20 genes related with GAS5 in kidney renal clear cell carcinoma [52].

**Table 2:** Predicted GAS5 targets with ceRNA mechanism of action. Shown are target genes of GAS5 whose expression could be modeled using putative ceRNA regulators yielding R^2^ of 0.2 or greater, with an empirical p-value < 0.01, i.e., where none of the 100 modeling attempts using equally many random lncRNAs yielded a better R^2^. (* GAS5 target genes which are also included in the top 20 genes related to GAS5 in [27]).

mRNA	Test *R*^2^	OLS p-value of GAS5
RPS18 *	0.39	2.23E-37
RPL31 *	0.35	1.06E-23
RPL15	0.33	7.20E-04
RPL30 *	0.33	1.05E-28
RPL5 *	0.30	4.21E-09
RPS4X	0.28	7.46E-09
SLC25A6	0.27	7.94E-06
RPS20	0.27	2.07E-12
RPL24	0.25	1.13E-61
RPS15A	0.24	4.80E-06
RPL34 *	0.24	2.91E-58
LY6G5B	0.23	7.67E-11
RPS12	0.22	9.69E-04
RPSA	0.21	1.11E-12
RRM2B	0.21	2.47E-14
EIF1	0.21	2.08E-03
ABCF1	0.21	2.51E-03
TRAF2	0.20	3.40E-07

In summary, we found multiple lines of evidence suggesting cis action as a dominant mechanistic theme in lncRNA-driven regulation, but relatively limited evidence in supporting of the trans-acting ceRNA mechanism. (Also see Discussion.)

### Statistical interaction analysis reveals potential protein mediators of lncRNA action

Beyond cis-regulatory and ceRNA mechanisms, lncRNAs are also known to function by acting as scaffolds for RNA- and/or DNA-binding proteins [34]. We hypothesized that such mechanisms may be detectable in the current data if we simultaneously examine expression variations of a target gene (mRNA), candidate lncRNA regulators and (mRNA of) potential protein mediators. Here, we report our findings from such an analysis where we detected significant statistical interactions between lncRNAs and either TFs or RNA-binding proteins (RBPs) in modeling mRNA expression.

*Interactions with TFs*: Above, we used a set of ~10 automatically selected lncRNAs and, separately, a set of ~10 selected TFs to model each target mRNA (**Fig 2F** and **Fig 2D** respectively). We now included both sets as covariates and re-trained a linear model to predict the target mRNA’s expression across all 1,217 samples as a function of these predictors. Starting with this combined “lncRNA + TF predictors” model as a baseline, we tested each lncRNA-TF pair for evidence of interaction by including their product as an additional covariate, re-training the model and extracting the statistical significance of the interaction term. Such interaction terms were added to the model iteratively, using the Akaike Information Criterion (AIC) as a measure of improved fit (Step-AIC method [53]). Inclusion of interaction terms improved the R^2^ values achieved by the linear model, as expected, but the AIC criterion assured us that improvements were not merely due to additional free parameters. The median R^2^ value achieved over all target mRNAs was now at 0.69, compared to 0.67 (t-test p-value = 2.68e-27) from the baseline model without interactions. Summarizing all significant interactions (**S7–S10 Tables**, limited to target genes whose R^2^ values were above median), we noted that the TF SPI1 is involved in the most interactions (88, compared to a median of 5, see **S11 Table**). This is consistent with literature reports of this TF being associated with several lncRNAs [54–56]. We further focused on significant lncRNA-TF interaction terms where the target gene is differentially expressed (t-test p-value < = 0.05) between samples with high (above median) lncRNA and TF levels compared to samples that have low (below median) levels of either putative regulator. These 1,058 short-listed terms (**S12 Table**) present putative cases where the simultaneous presence of lncRNA and TF has an effect on target gene expression. In **Fig 3F**, we present the case of an interaction term involving the lncRNA TMPO-AS1 and the TF EBF1 (Bonferroni corrected p-value 3.23E-10), used in modeling the expression of gene LRP1. TMPO-AS1 has been implicated in several cancers, is activated by EBF1 and also indirectly activates EBF1 by sponging the microRNA miR-98-5p in bladder cancer [57]. Moreover, the predicted target, LRP1, is a repressor of the Wnt signaling pathway [58] and TMPO-AS1 has been shown to activate the Wnt pathway [59]. These observations together suggest that a repressive effect of TMPO-AS1 on LRP1 in the presence of TF EBF1 underlies the lncRNA’s activating influence on the Wnt pathway.

*Interactions with RBPs*: We performed a similar analysis to systematically detect lncRNA-RBP statistical interactions in prediction of mRNA expression (**S13 Table**). The inclusion of RBP interaction terms significantly enhanced the performance of the model, increasing the median R-squared value from 0.29 to 0.33 (t-test p-value: 1.51e-199). Summarizing all significant interactions (**S14–S16 Tables**, limited to target genes whose R^2^ values were above median), we observed the RBPs ESRP1 and ESRP2 to be among the top four RBPs by frequency of detected interactions. ESRP1 has been reported as controlling ER+ breast cancer [60], and ESRP1/ESRP2 were noted as being the most strongly down-regulated RBPs in epithelial-to-mesenchymal transition (EMT) in a cell culture model of breast cancer [61]. The most frequent RBP (by frequency) per our analysis–PCBP1 –has also been shown to regulate EMT in breast cancer [62]. Next, as for TFs, we next short-listed 2,425 interaction terms with the above-mentioned pattern of differential target gene expression between categories of samples defined by high and low expression of lncRNA and RBP (**S17 Table**). One such term involves the lncRNA OIP5-AS1 and the RBP FXR1, with a p-value of 7.8E-17 when used for modeling the target gene MYL12A. As shown in **Fig 3G,** samples with high levels of the lncRNA and the RBP have significantly higher expression of the target gene MYL12A than those with low levels of either regulator, especially the lncRNA. Indeed, OIP5-AS1 has been shown to bind to FXR1 in order to regulate other genes, activate the Wnt signaling pathway and contribute to cell proliferation [63,64]. Another example is the interaction term for lncRNA SNHG11 and RBP SNRPB2, found significant when modeling the target gene RPRD1B (Bonferroni corrected p = 5.5E-17). We noted that the lncRNA (SNHG11) is located in cis to (< 400 Kbp separated from) the target gene (RPRD1B), adding support to the regulatory relationship. We also examined the interaction terms associated with the lncRNA MALAT-1, which is among the best studied lncRNAs in breast cancer context [22,65]. Our analysis (**S17 Table**) finds 12 such RBPs, including SRRM1, SRRM2, and SRSF1, which have been shown to interact with MALAT-1 [46,66].

To summarize, extending the linear model for gene expression to include interaction terms allowed us to generate hypotheses regarding potential mechanisms by which a lncRNA may exert its regulatory influence, mediated by or while competing with RNA binding proteins or transcription factors.

### A multi-evidence lncRNA regulatory network

**Fig 4:**
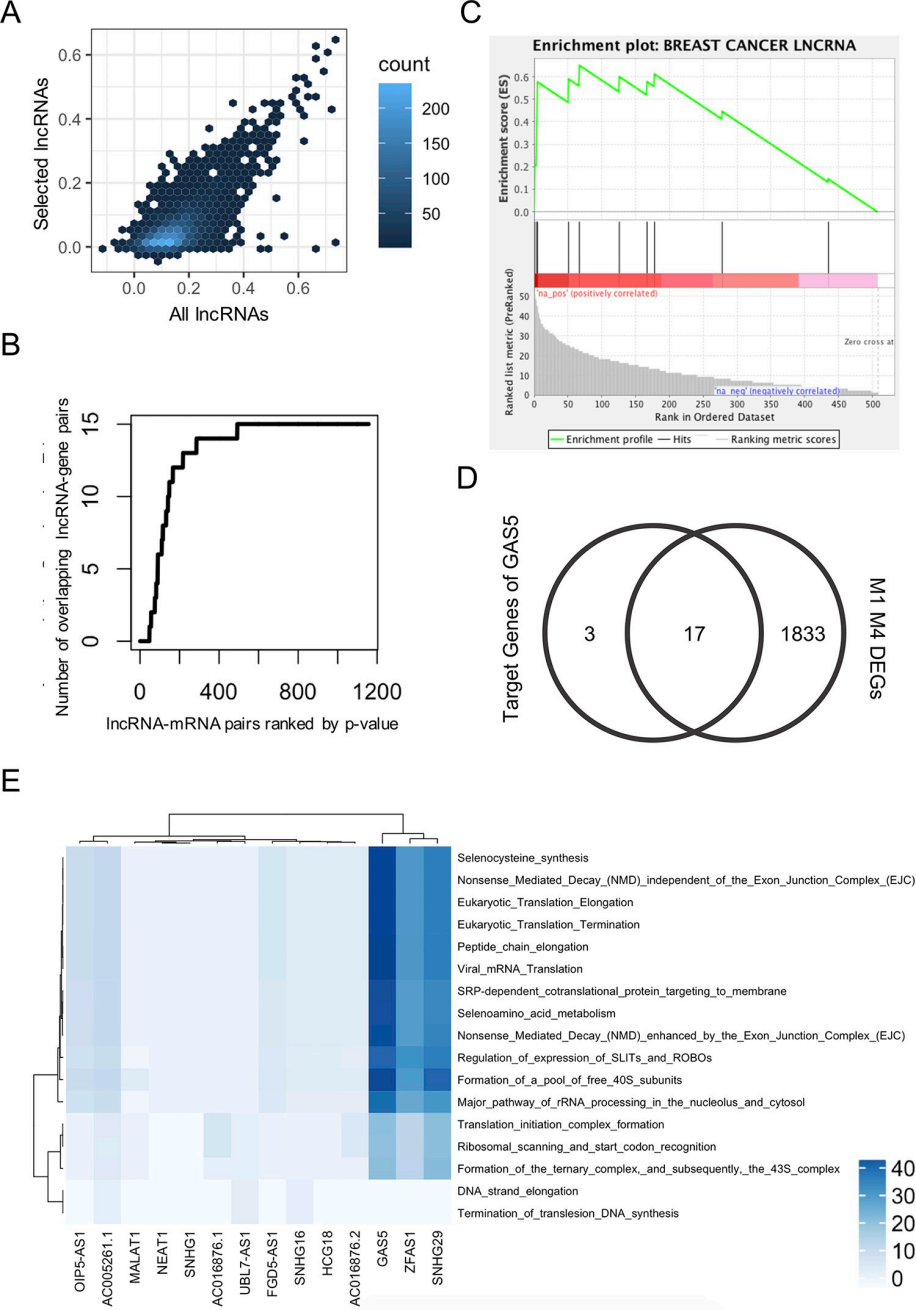
Expression modeling and regulatory network using selected lncRNAs with location- or mechanism-based supporting evidence. (A) *R*^2^ values using selected lncRNAs (on average ~2 lncRNAs per target gene) were comparable to those obtained when utilizing all lncRNAs as candidates. (B) Network edges (lncRNA-gene pairs) in the “overlapping” category are strongly enriched among the edges with the strongest p-values from expression modeling. X-axis represents the network edges sorted by p-value from expression modeling, and Y-axis shows the number of “overlapping” lncRNA-gene pairs among the top x network edges, for each value of x. (C) Breast cancer related lncRNAs are enriched among lncRNAs with high degrees in the regulation network. Shown is the standard graphical output from the GSEA tool. X-axis represents lncRNAs sorted by the number of putative gene targets (edges in the network). Breast cancer-related lncRNAs from the literature are marked by vertical bars in the middle panel, and the top panel (green curve0 shows the “enrichment score” statistic calculated by GSEA as a function of the rank order. This analysis shows that breast cancer-related lncRNAs tend to have significantly more targets compared to other lncRNAs, in our network. (D) Venn diagram of predicted GAS5 targets that are also differentially expressed genes (DEGs) between M1 and M4 cell lines. (E) Target genes of four high-degree lncRNAs (more than 25 targets each) were enriched in REACTOME pathways related to translation processes.

In the analyses above, we relied on expression modeling to identify potential lncRNA regulators of a gene, and then annotated them with features such as cis-location, ceRNA, or statistical interactions with proteins, that provide supporting evidence or plausible mechanisms for a subset of these regulators. Our final goal was to integrate these two complementary sources of information: to model each target gene using only the small set of candidate lncRNA regulators for which there exists supporting evidence found in the various annotation steps above. Specifically, we limited the candidate regulators for each target gene to those that were statistically significant in above-trained models for that gene (p-value of OLS coefficient < = 0.01) and had been annotated with any of the location categories of “overlapping”, “same-TAD”, “proximal” (**S4 Table**) or the mechanistic category of “ceRNA” (empirical p-value = 0, **S6 Table**) or had been found in a statistical interaction term involving a TF (**S7 Table**) or an RBP (**S13 Table**). As a result, for each target gene there were about two candidate lncRNAs, which were used in an ordinary least square regression to fit the gene’s expression profile. The R^2^ values in this exercise were comparable to those obtained when utilizing all lncRNAs as candidates (**Fig 4A**), even though the number of candidates was two orders of magnitude smaller, with 271 (5.0%) of the 5,364 genes modeled meeting the R^2^ > = 0.2 threshold. We focused on these target genes (mRNAs), constructing a regulatory network comprising their significant lncRNA predictors (p-value of coefficient < = 0.01). This network (**S18 Table**) includes 508 lncRNAs regulating 232 mRNAs, a total of 1157 lncRNA-mRNA regulatory relationships, with an average of 2.3 targets per lncRNA and 5.0 regulators per mRNA. We calculated the correlation between the lncRNA regulator and the mRNA target for the edges in the predicted network and, separately, for 1157 randomly sampled pairs, using RNA-seq data for 54 tissues from GTEx (**S22 Table**). The edges have significantly higher correlation compared to the random pairs in breast mammary tissue with t-test p-value of 2.1e-10, suggesting significantly higher co-expression for predicted lncRNA-mRNA regulatory pairs. Only 199 edges would be detected by the co-expression based regulatory network from the same transcriptomic dataset where 1157 most correlated lncRNA-mRNA pairs among the same 508 lncRNAs and 232 mRNAs are defined as lncRNA-mRNA associations. In the co-expression network, each lncRNA has 4.2 targets on average, which is significantly higher (p-value: 1.04e-7) than the average number of targets per lncRNA in the regulatory network, suggesting that co-expression-based approaches might be in favor of common regulators whose expression are highly correlated with many targets and fail to find regulatory lncRNAs with more specific function.

We further validated a subset of the putative lncRNA-mRNA pairs using experimentally measured chromatin interaction from GRID-seq assays on the breast cancer epithelium cell line MDA231 [67,68]. These data provided RNA-DNA interaction information for 23 of the lncRNAs present in the multi-evidence network. The network includes 111 edges involving these lncRNAs, 29 (26.1%, hypergeometric test p-value: 5.7e-8, see Methods) of which are supported by the GRID-seq data as interactions between lncRNA and proximal region (1 Mbp) of the target gene (**S18 Table** Column “GRID-seq”). Notably, all of the nine predicted edges involving MALAT1 and six of the seven predicted targets of NEAT1 are supported by GRID-seq data.

The network included 549 ceRNAs and 514 cis-acting (overlapping, same-TAD or proximal) lncRNAs. We noted a clear enrichment of the “overlapping” category among the edges with the strongest p-values from expression modeling (**Fig 4B**). Each edge is supported by some form of evidence, e.g., location near or in cis with target gene, ceRNA evidence, statistical interaction with a TF or RBP, in addition to its expression being predictive of the target’s expression, making this a higher confidence, multi-evidence lncRNA regulatory network. Among the 388 lncRNAs with cis-acting edges in the network, 271 overlap the regulatory genetic regions of human mammary epithelial cells obtained from GenenHancer [69]. Given that 745 of the 1074 lncRNAs analyzed in this work overlap the regulatory regions, the lncRNAs reported with cis-edges in the network showing no enrichment for enhancer marks (Odds ratio: (271/388)/(745/1079) = 1.01, p-value:0.64), suggesting cis-acting lncRNAs reported in the network don’t merely mark active regulatory regions.

We observed 53 of the 508 lncRNAs in this network to have 25 or more targets (> 10% of the 232 target mRNAs in the network, see **S19 Table**) and significantly higher expression compared to other lncRNAs (p-value: 1.92e-6), suggesting a possible prioritization of lncRNAs with the most regulatory role. Indeed, these “hubs” included several lncRNAs well known for their role in breast cancer, e.g., MALAT1 [22,65] at rank 17, SNHG29 [70] at rank 2, SNHG16 [71] at rank 19, GAS5 [50,72] at rank 1, XIST [73,74] at rank 11, NEAT1 [75,76] at rank 23, NORAD [17,77] and others. (It is noteworthy that the majority of connections associated with these lncRNAs are predominantly trans-regulatory interactions, see **S21 Table** and **Fig I in S1 Text**) Pursuing the above observation systematically, we obtained 30 breast cancer-related lncRNAs from the LncRNADisease database [72], of which 10 were included in the high confidence regulatory network. Four of these 10 were among the 53 high-degree regulators noted above, and a Gene Set Enrichment Analysis (GSEA) revealed a significant association (p-value < 0.027) between the breast cancer-related lncRNAs and the degree of a lncRNA in the network, as shown in **Fig 4C**.

Next, we functionally characterized the predicted targets of each high-degree regulator (lncRNA) through gene set analysis. We first conducted enrichment tests against a previously published set of genes that are differentially expressed (DE) between benign (“M1”) and highly tumorigenic (“M4”) states of a widely used breast cancer progression cell line series [18] derived from MCF10A mammary epithelial cells. We found two of the lncRNAs—GAS5 and SNHG29—to have targets significantly enriched in the M1-M4 DE genes (FDR < 0.05), with Hypergeometric test p-value 5E-6 (**Fig 4D**) and 6E-4 respectively. Expanding the enrichment tests to iterate over an entire compendium of biological pathways from the REACTOME database [78], we observed a set of four lncRNAs (OIP5-AS1, GAS5, ZFAS1, SNHG29) to have their targets enriched for several pathways related to translation processes (**Fig 4E**, **S20 Table,** and **Fig J in S1 Text**). For instance, the lncRNA GAS5, a tumor suppressor that is down-regulated in mammary carcinoma [79], has 20 targets in the lncRNA-mRNA network and 12 of these putative targets belong to the pathway “peptide chain elongation” (Bonferroni corrected p-value 3.23e-15). Indeed, this lncRNA is believed to regulate translation of specific proteins such as c-Myc by interacting with the translation initiation complex protein eIF4F [80], which is one of its predicted targets in our network.

The regulatory network comprises edges with directionality, i.e., not only is each lncRNA-mRNA relationship assigned a significant p-value in expression modeling, the sign of the coefficient in the linear model tells us if the relationship is activating or inhibitory. We noted 782 activating and 355 inhibitory edges overall in the network and identified four lncRNAs with a significant departure from the global ratio in one direction or the other (Binomial test p-value < 0.01, see **Figure H in S1 Text**). These included the lncRNA SNHG29, that has 19 targets in the network, of which 18 (94.7%) represent activating relationships.

While the majority of lncRNAs were initially thought to lack protein-coding potential, emerging evidence suggests that certain lncRNAs can indeed produce functional peptides [1]. These peptides, often referred to as "micropeptides," are short sequences of amino acids encoded by specific regions within lncRNA transcripts. They can be translated from non-canonical open reading frames (ORFs) and have been shown to have regulatory roles in various biological processes. Micropeptides derived from lncRNAs can associate with ribosomes and be involved in translation regulation or other ribosome-associated functions.

Overall, while the majority of lncRNAs are non-coding, there is growing evidence supporting a connection between certain lncRNAs and the production of ribosome-associated peptides, expanding the functional repertoire of these RNA molecules beyond their non-coding roles.

In summary, the regulatory network constructed here is based on both expression modeling and prior evidence of plausible mechanisms (cis-action, ceRNA or protein-mediated action), and we found several additional lines of evidence supporting its compendium of regulatory relationships: enrichment of the highest degree regulators for reported involvement in breast cancer, association of target gene sets with DE genes from a breast cancer progression cell line series and with translation-related as well as other relevant biological processes, and statistically significant biases towards either activating or inhibitory relationships.

## Discussion

This study was aimed at systematically studying lncRNAs’ impact on gene expression variation among individuals in a breast cancer cohort, in the hope of teasing apart potentially causal effects from chance correlations, reaching statistical estimates of the extent to which gene expression may be under lncRNA regulation, and taking the first steps towards lncRNA regulatory network reconstruction considering both expression correlation and potential regulation mechanisms. Importantly, the specifics of our findings here, such as percentage of variation explained or the predicted regulatory interactions, need not generalize to other biological contexts and further work is needed to assess the extent of their generalizability and context-specificity. We performed the study on data from a cohort of breast cancer tumor samples, in part because of easy availability of data (over a thousand samples) and partly because of the emerging significance of lncRNA regulation in cancer [81–84]. In comparison to prior work reporting on co-expression networks derived from pairwise (lncRNA-gene) correlation analysis [8,9,11], we adopted a strategy of multivariable regression of target genes using lncRNAs as predictors, borrowing inspiration from how transcriptional regulatory networks (TRNs) of TF-gene relationships are commonly reconstructed today [26,29,85]. While linear as well as non-linear models have been successfully used in TRN reconstruction, we adopted linear models for ease of interpretability and ability to estimate significance of individual predictors. We used regularization (Elastic Net) to ensure that each gene is modeled using a small selection of candidate lncRNA regulators and relied on cross-validation to assess how well target gene expression can be modeled using lncRNAs.

An important feature of our methodology was to treat transcription factors as potential confounders for regulatory modeling and limit the assessment of lncRNA-driven regulation to the gene expression variation that cannot be explained using TFs. It is likely that this step removed part of the expression variance caused by lncRNA regulation, but we chose to be conservative with lncRNAs’ regulatory impact, given that TFs, compared to lncRNAs, are better studied gene expression regulators and expression-based models of TF-gene regulatory networks are an accepted strategy to quantifying their effects. We did not remove the influence of shared RBPs, which can also act as confounders for lncRNA-mRNA relationships, because RBP-mediated regulation is a potential mechanism through which a lncRNA may regulate the expression of the target gene. Regressing out the impact of RBP from mRNA expression might lead to the failure to identify regulatory lncRNAs whose function depends on RBP binding.

We found that for over 16% of genes analyzed, lncRNAs can explain more than 20% of expression variation, even after accounting for possible co-regulation by TFs. We performed various statistical assessments and comparisons of this number. For instance, repeating a similar analysis with an equally large set of enzyme-encoding mRNAs in place of lncRNAs as regulators, we could establish a null distribution that yielded an empirical p-value of 0.01 for the lncRNA-driven regulation of over 6% of analyzed genes. (Such an extent of inferred regulatory effect is expected by chance for only 1% of genes.)

Using simple criteria to filter the regulators identified during regression modeling, we obtained 643 high confidence lncRNA-gene regulatory relationships, and though these were identified in an unbiased manner, purely from expression data, we observed a clear tendency for the regulator lncRNA to be located in ‘cis’ to the gene. For instance, for 27.5%, 35.5%, 54% and 62.7% of the above pairs, the lncRNA is overlapping, located in the same TAD as, proximal (1 Mbp) to, or on the same chromosome as the target gene, respectively (**Table 1**). Using suitable controls, we confirmed that these percentages are not merely a reflection of cis-located gene pairs having a tendency for co-expression.

Furthermore, by repeating the regression analysis using only the lncRNAs from a specific location category (overlapping, same-TAD, proximal, or same chromosome) as predictors, we were able to assess the overall regulatory importance of each category (**Fig 3E**). We noted that over 16% genes that have an overlapping lncRNA can be modeled well using that lncRNA, indicating lncRNA-gene overlaps as a reliable marker of regulatory function. About 20% of lncRNA-harboring TADs were found to include at least one gene that can be modeled well using lncRNAs in the same TAD (compared to 8.8% by chance). Similarly, about 8% of all genes could be modeled well using lncRNAs from the same chromosome. Thus, while ~1,800 of analyzed genes could be modeled well using an unrestricted choice of lncRNAs, about 50% of these are amenable to expression prediction using only (putatively) cis-acting lncRNAs from the same chromosome. While we found multiple lines of evidence in favor of cis-action by lncRNA regulators, we did not observe nearly as much influence belonging to the trans-acting, competing endogenous RNA (ceRNA) mechanism. For instance, among the 643 lncRNA-gene pairs identified in an unbiased manner, only 19 (~3%) were plausible ceRNAs. Similarly, when repeating the modeling of target gene expression using only ceRNAs, only ~2% (246 out of 10556) of analyzed genes could be modeled well. These observations may point to shortcomings of the ceRNA compendium we relied on (derived from experimental miRNA binding data but based on computational predictions of competing lncRNA-miRNA and mRNA-miRNA pairs) or to unknown methodological limitation that limits our ability to detect ceRNA relationships from expression data alone.

The functional role of competing endogenous RNAs (ceRNAs) has been extensively investigated, with reports of numerous lncRNA-miRNA-mRNA triplets, and led us to employ it as a plausible regulatory mechanism in our study. However, there have also been doubts raised regarding the extent of their influence [86], and our findings of limited predictive value of this class of candidate regulators raise similar doubts. The low predictive value may also be attributed to the fact that ceRNA candidates used in our models have relatively limited prior support, being based on computational predictions of competition for common miRNAs; our working definition–the shared targeting of lncRNAs and mRNAs by the same miRNA within the same cell line–does not guarantee the formation of definitive ceRNA triplets. As experimentally verified ceRNA pairs become available in the future, their integration with our analysis holds the promise of enhancing the accuracy of predicting lncRNAs’ trans regulatory effects.

It is believed that lncRNAs may interact with TFs and RBPs as scaffolds as part of their regulatory activity. Motivated by this, we statistically analyzed the significance of interaction terms involving lncRNA-TF pairs or lncRNA-RBP pairs as additional features in modeling a target gene’s expression. This helped us identify thousands of cases where a lncRNA’s regulatory effect may depend on the expression level of another regulator such as a TF or an RBP (**S12** and **S17 Tables**). The TF SPI1 and the RBPs ESRP1, ESRP2 and PCBP1 featured frequently in detected statistical interactions, suggesting additional mechanistic roles for these proteins in breast cancer.

Finally, we created a high confidence network of lncRNA regulatory influences, where the lncRNA is not only a significant predictor of target gene expression but is also located proximally to the gene, or is potentially supported by an RNA or DNA-binding protein, or is a putative ceRNA (**S18 Table**). Top regulators in this network are enriched for breast cancer-related lncRNA (**Fig 4C**) and their targets are enriched for genes differentially expressed in breast cancer progression (M1-M4 cell lines). Interestingly, several of the top regulators have a significant bias in the sign (positive or negative) of co-expression with their targets (**Fig H in S1 Text**), further supporting their regulatory roles since chance co-expression of a lncRNA with dozens to hundreds of genes is not expected to have a significant bias in directionality of correlation.

Our analyses were performed using RNA-seq data from TCGA, obtained using an earlier processing pipeline that relied on the “HTSeq-count” tool to count reads mapping to genes. This tool, now superseded by STAR-2, may map the same reads to multiple overlapping genes, which may potentially affect our discovery of statistical relationships between overlapping lncRNA-mRNA pairs. However, when we re-calculated lncRNA-mRNA expression correlations using the up-to-date expression quantification data that address the above issue, we found the correlations to be largely in agreement with corresponding correlations from the previous version of TCGA data used in our study (see **Fig K in S1 Text**). This was true for the entire collection of lncRNA-mRNA pairs deemed as putative regulatory pairs in our work, including for overlapping pairs. This suggests that the lncRNA regulatory compendium reported by us should remain largely unaffected by the transition of TCGA processing pipelines.

Our work is a first step towards systematic reconstruction of lncRNA regulatory networks. The variety of mechanisms that have been reported for lncRNA-driven regulation make this an exciting challenge, and we expect future efforts to integrate available information on such mechanisms, such as ceRNAs, cis-action, protein-mediated mechanisms, etc., into the network inference. Methods for utilization for mechanistic prior information have been successfully demonstrated in the context of TF regulatory networks [28] and will be a key strategy towards enriching lncRNA-gene co-expression networks for causal relationships. Such networks can furnish testable hypotheses regarding specific lncRNA regulators of a gene of interest and also prioritize lncRNA with extensive global regulatory roles in a biological process, ultimately providing a more complete picture of the functions of this important class of non-coding RNAs.

## Methods

### Expression data collection and pre-processing

Transcriptome profiling data from 1092 patients with breast invasive carcinoma were obtained from The Cancer Genome Atlas (TCGA) breast cancer cohort (TCGA-BRCA project). The dataset consisted of RNA-Seq expression measurements and clinical data for 1217 primary tumor samples. The HTSeq-counts data encompassed measurements for 60488 RNAs on the gene level. RNA-Seq data processing steps are documented at the GDC portal (URL https://docs.gdc.cancer.gov/Data/Bioinformatics_Pipelines/Expression_mRNA_Pipeline/). To ensure reliable analysis, RNAs with counts per million (CPM) greater than 1 in fewer than 500 samples were removed due to low expression, resulting in a filtered expression dataset containing 15766 RNAs with adequate expression levels. RNA annotations were retrieved from the Ensembl database (Ensembl 104: May 2021) [87]. The filtered matrix comprised expression data for 13963 protein-coding genes and 1079 long non-coding RNAs (lncRNAs). Note that since TCGA RNA-seq data collection involved a polyA enrichment step, lncRNAs that are not polyadenylated will not be suitable represented in the analyzed data. The raw HTSeq-counts were normalized using TMM normalization in the R package “EdgeR”. The TMM-normalized expression values were then log-transformed and standardized to have zero mean and variance equals to 1. Confounder variables including age, sex, ethnicity and race for patients were obtained from the clinic data for the same cohort. The transformed normalized expression values of all RNAs were modeled using the confounder variables via simple linear regression models and residuals from these confounder-based models were used for further modeling.

### Regression models of expression data

We trained linear models to predict each gene’s expression using different sets of predictors, which included lncRNAs, TFs or confounder variables. ElasticNet algorithm was used to select ~10 predictors (features) when there were more than 10 candidates. An ordinary linear squares (OLS) regression model was then trained using the selected features. For genes with fewer than 10 predictors, an OLS model was used directly to predict the gene’s expression using all the predictors, without any feature selection. For most analyses here, 5-fold cross validation was used and test *R*^2^ averaged across the five folds was used to measure the performance of the model. After cross validation, the same process (ElasticNet followed by OLS model) was repeated on the entire data set and the resulting *R*^2^ was reported as the training or “overall” *R*^2^.

### Transcription factors as confounders

We obtained identities of 1665 human transcription factors (TFs) from HumanTFDB [88]. Expression profiles for 1255 of these TFs were available in the expression data set. We first used Elastic Net algorithm to select ~10 TF regulators to predict the expression of each gene. If the gene being modeled is a TF itself, it was removed from the predictor set when training the Elastic Net model. The gene’s expression was then modeled using the selected TFs. Residuals from the TF-based model were then subjected to modeling using lncRNA predictors as described above.

### Statistical lncRNA-RBP interaction analysis

We first modeled mRNA residuals from the TF-based models using ~10 RNA-binding protein (RBP) genes selected by the Elastic Net algorithm out of a candidate set of 351 RBPs obtained from RBPDB V1.3.1 [89]. We then used the candidate set of ~10 lncRNAs and ~10 RBPs to model each mRNA’s residual from the TF-based model. The model with the lncRNA and RBP predictors forms the baseline model for each mRNA. We then identified significant lncRNA-RBP interaction terms iteratively using the Step-AIC method [53]. We reported the *R*^2^ values from the lncRNA-based models, RBP-based models, lncRNA+RBP (baseline) models and lncRNA-RBP interaction models.

### Location and mechanism categorization of lncRNAs

We categorized putative lncRNAs based on the following relationships to target genes: 1) Overlapping: LncRNA overlaps the gene on either DNA strand. Locations of protein coding genes and lncRNAs were obtained from Ensembl BioMart. 2) Same TAD: TAD boundaries from Hi-C data for MCF-10A mammary epithelial cell line were used to define TADs [45]. LncRNAs in the same TAD with the gene were defined as “same TAD” lncRNAs. 3) Same Chromosome: LncRNAs in the same chromosome as the target gene were included in the “same chromosome” category. 4) ceRNAs: Experimentally supported miRNA-gene interactions were downloaded from TarBase v.8 [47]. Relationships between miRNAs and lncRNAs were obtained from lncBase v.2 [90]. LncRNAs that are related to the same miRNAs as the target gene in the same cell lines were considered to be the competing endogenous RNAs (ceRNAs).

### GRID-Seq enrichment test

RNA-chromatin interactions in the breast cancer epithelium cell line MDA231 were obtained from GRID-seq profile GSE82312. Twenty three lncRNAs are measured in both GRID-seq and TCGA RNA-seq data. Hypergeometric test was used to measure the overall enrichment of lncRNA interacted genes in predicted edges:

1. Universe: size 318849 = (23 lncRNA included in both our study and GRID-seq) X (13963 genes included with TCGA RNA-seq measurements)
2. Set A: size 27757 = lncRNA-mRNA interaction pairs identified by GRID-seq, through binding evidence of lncRNA on 1mb region around the gene.
3. Set B: size 111 = predicted edges for 23 lncRNAs included in both studies
4. Intersection between Set A and Set B: size 29.
5. P-value: 5.738724e-08

### Pathway enrichment test

For lncRNAs with more than 25 putative target genes, associations between the target gene set and pathway gene sets from Reactome database [91] were detected via hypergeometric test of overlap between the two gene sets and FDR-corrected p-values were reported.

## Supporting information

S1 Table mRNA-lncRNA pairs satisfying the following properties: (1) the mRNA’s expression can be accurately (R2 > = 0.5) modeled using lncRNAs, (2) the R2 using lncRNA predictors is at least 0.2 greater than the R2 using TF predictors, (3) the lncRNA was one of the ~10 selected predictors of the mRNA’s expression and (4) its coefficient in an OLS model of the mRNA’s expression was assigned a p-value < = 0.05. Gene.ID, Gene.Name: identity of target mRNA. lncRNA.ID, lncRNA.Name: identity of predictor lncRNA. OLS.p-value: Statistical significance of coefficient of lncRNA as a covariate in an OLS model of target mRNA.
(XLSX)

S2 Table Number of times each lncRNA occurs as a significant predictor in S1 Table. (XLSX)

S3 Table Target genes (mRNA) whose expression could be modeled accurately (R2 empirical p-value < = 0.01) using lncRNAs. Empirical p-value was computed for each mRNA by comparing the R2 value obtained using lncRNAs as predictors to R2 values obtained using enzyme-encoding RNAs as predictors. 100 replicates of the “control” using enzyme-encoding RNAs were performed, allowing us to infer empirical p-values at a resolution of 0.01. The 699 genes (mRNAs) reported here comprise 6.1% of the 11,531 target genes analyzed.
(XLSX)

S4 Table Details of 643 mRNA-lncRNA pairs meeting the following criteria: the mRNA can be modeled using lncRNAs with R2 > = 0.2 and empirical p-value < 0.01, the lncRNA has an OLS p-value < = 1E-10 in the linear model for the mRNA, and their pairwise correlation coefficient is > = 0.1 in absolute value. Gene.ID, Gene.Name: identify of target gene (mRNA); lncRNA.ID, lncRNA.Name: identity of lncRNA; OLS P-value: p-value of coefficient of lncRNA in OLS model of mRNA; R2: test R2 of model, averaged over five folds of cross-validation; Columns G-K indicate relationship between mRNA and lncRNA; Columns L-N indicate if an overlapping pair is tandem (same direction), convergent or divergent; dist: distance between lncRNA and mRNA genes, -1 means overlapping, ‘X’ means not on the same chromosome; Column P (“Corr Coeff”) is Pearson correlation coefficient between mRNA and lncRNA expression.
(XLSX)

S5 Table Statistical assessment of degree of correlation seen in lncRNA-mRNA pairs defined by various criteria of relative location (“category”). For each category, a random set of lncRNA-mRNA pairs belonging to that category was first selected, without regard to their expression profiles. Column B shows the size of this random set and Column C shows the number of members of this set for which the Pearson Correlation coefficient (PCC) in expression profiles was found to be > = 0.3. Next, we selected a random set of mRNA-mRNA pairs belonging to the category (e.g., mRNA pairs located in the same TAD if the category is “same-TAD”) as a background set; columns D and E respectively show the size of this random set of mRNA pairs and the subset thereof for which the mutual expression correlation (PCC) is > = 0.3. The latter two numbers allow us to define a baseline frequency of co-expression (PCC > = 0.3) between two genes related to each other by a particular definition (e.g., in the same TAD), which is then used to assess the statistical significance (Binomial p-value) of the number of lncRNA-mRNA pairs reported in Column C.
(XLSX)

S6 Table Target genes and R2 values from models using putative ceRNAs as regulators. R2 values are average of test R2 from five folds of cross validation. Empirical p-values were calculated by repeating the modeling 100 times using random sets of lncRNAs as candidate regulators.
(XLSX)

S7 Table All statistically significant (Bonferroni corrected p-value < = 0.05) lncRNA-TF interaction terms detected when modeling expression of a gene (Gene.id, Gene.name) using a linear model with selected number of lncRNA and TF covariates as well as an interaction term involving the lncRNA and TF. (XLSX)

S8 Table Frequency lncRNA-TF pairs detected as statistically significant terms. “Count” represents the number of target genes for which the particular lncRNA-TF pair had a significant interaction term.
(XLSX)

S9 Table Frequency with which each lncRNA appears among significant lncRNA-TF interaction terms. For each lncRNA (lncRNA.id, lncRNA.name), Column C (#gene) shows the distinct target genes for which an interaction term involving this lncRNA and some TF was statistically significant; Column D (#tf) shows the number of distinct TFs represented in these interaction terms. (Note: the same lncRNA, paired with the TF, may be significant for more than one target gene, and the same target gene may have more than one interaction terms involving the same TF.)
(XLSX)

S10 Table Frequency with which each TF appears among significant lncRNA-TF interaction terms. For each TF (TF.id, TF.name), Column C (#gene) shows the distinct target genes for which an interaction term involving this TF and some lncRNA was statistically significant; Column D (#lncRNA) shows the number of distinct lncRNAs represented in these interaction terms.
(XLSX)

S11 Table All significant interaction terms involving the TF SPI1. For each such interaction term, the target gene is reported in columns A,B (gene.id, gene.name), the interacting lncRNA in columns C,D (lncrna.id, lncrna.name), and the Bonferroni corrected p-value for the interaction term involving SPI1 (TF) and that lncRNA in prediction of the target gene is shown in Column G.
(XLSX)

S12 Table Selection of significant (target gene, lncRNA, TF) triplets such that the lncRNA-TF pair forms a statistically significant (Bonferroni corrected p-value < 0.01) interaction term in modeling the target gene’s expression. Columns A-F indicate the id and name of the target gene, lncRNA and TF in that order, while column G reports the Bonferroni corrected p-value for the interaction term. Columns H-Q report on the p-value of differential expression of the target genes between two groups of samples defined as: HH = samples with high levels of lncRNA and high levels of TF; HL = samples with high levels of lncRNA and low levels of TF; LH = samples with low levels of lncRNA and high levels of TF; LL = samples with low levels of lncRNA and low levels of TF. Thus columns labeled as HH-LH report on differential expression (of target gene) between the HH and LH groups, etc. For each contrast, column with label suffixed with “.direction” reports on whether the average gene expression is higher (1) or lower (-1) in the first group compared to the second group; column with label suffixed with “.p” reports the p-value from a T-test between the two groups. The selection of triplets shown here meet the additional criteria that the HH-HL, HH-LH, and HH-LL contrasts are statistically significant (p-value < 0.05) and have the same direction of differential expression (all 1 or all -1).
(XLSX)

S13 Table All statistically significant (Bonferroni corrected p-value < 0.01) lncRNA-RBP interaction terms detected when modeling expression of a gene (Gene.id, Gene.name) using a linear model with selected number of lncRNA and TF covariates as well as an interaction term involving the lncRNA and TF. (XLSX)

S14 Table Frequency lncRNA-RBP pairs detected as statistically significant terms. “Count” represents the number of target genes for which the particular lncRNA-RBP pair had a significant interaction term.
(XLSX)

S15 Table Frequency with which each lncRNA appears among significant lncRNA-RBP interaction terms. For each lncRNA (lncRNA.id, lncRNA.name), Column C (#gene) shows the distinct target genes for which an interaction term involving this lncRNA and some RBP was statistically significant; Column D (#rbp) shows the number of distinct RBPs represented in these interaction terms.
(XLSX)

S16 Table Frequency with which each RBP appears among significant lncRNA-RBP interaction terms. For each RBP (RBP.id, RBP.name), Column C (#gene) shows the distinct target genes for which an interaction term involving this RBP and some lncRNA was statistically significant; Column D (#lncRNA) shows the number of distinct lncRNAs represented in these interaction terms.
(XLSX)

S17 Table Selection of significant (target gene, lncRNA, RBP) triplets such that the lncRNA-RBP pair forms a statistically significant (Bonferroni corrected p-value < 0.01) interaction term in modeling the target gene’s expression. Columns A-F indicate the id and name of the target gene, lncRNA and RBP in that order, while column G reports the Bonferroni corrected p-value for the interaction term. Columns H-Q report on the p-value of differential expression of the target genes between two groups of samples defined as: HH = samples with high levels of lncRNA and high levels of RBP; HL = samples with high levels of lncRNA and low levels of RBP; LH = samples with low levels of lncRNA and high levels of RBP; LL = samples with low levels of lncRNA and low levels of RBP. Thus columns labeled as HH-LH report on differential expression (of target gene) between the HH and LH groups, etc. For each contrast, column with label suffixed with “.direction” reports on whether the average gene expression is higher (1) or lower (-1) in the first group compared to the second group; column with label suffixed with “.p” reports the p-value from a T-test between the two groups. The selection of triplets shown here meet the additional criteria that the HH-HL, HH-LH, and HH-LL contrasts are statistically significant (p < 0.05) and have the same direction of differential expression (all 1 or all -1).
(XLSX)

S18 Table Multi-evidence lncRNA regulatory network. Columns A,B indicate target gene, columns C,D indicate regulator lncRNA, column E indicates p-value of OLS coefficient. Columns F-L indicate the type of additional supporting evidence for the relationship. Column M indicate if the interaction is supported by GRID-seq data.
(XLSX)

S19 Table lncRNA regulon sizes in the multi-evidence regulatory network. Columns A,B indicate lncRNA, column C indicates the number of predicted regulatory targets (mRNAs) of that lncRNA.
(XLSX)

S20 Table REACTOME pathway associations for gene targets of each lncRNA in the multi-evidence regulatory network. Columns A,B indicate lncRNA, column C indicates the REACTOME pathway, Columns D-G are the cardinality of universe, lncRNA regulon, pathway, overlap set respectively, with Column H and G being the Hypergeometric test p-value without and with Bonferroni correction.
(XLSX)

S21 Table lncRNA regulon sizes in the trans (ceRNA) regulatory network. Columns A,B indicate lncRNA, column C indicates the number of predicted regulatory targets (mRNAs) of that lncRNA.
(XLSX)

S22 Table Cross-tissue correlation of regulatory edges from the multi-evidence regulatory network (S18 Table) using RNA-seq data from GTEx. (XLSX)

S1 Text Figure A in S1 Text. Histogram of *R*^2^ values when mRNA expression was modeled using lncRNAs only. **Figure B in S1 Text.** Histogram of Pearson Correlation Coefficient between real and model-predicted expression values on test samples. **Figure C in S1 Text.** Density scatter plot of Pearson Correlation Coefficients between predicted and real expression values. **Figure D in S1 Text.** Histogram of overall *R*^2^ values when residuals from TF-based models were predicted using lncRNA. **Figure E in S1 Text.** Histogram of test *R*^2^ values modeling permuted expression. **Figure F in S1 Text.** Histogram of test *R*^2^ using overlapping lncRNAs only. **Figure G in S1 Text.** Scatter plot of test *R*^2^ values of models using all lncRNAs and those using overlapping lncRNAs only. **Figure H in S1 Text.** Comparison of negative targets and positive targets. **Figure I in S1 Text.** Comparison of cis targets and trans targets. **Figure J in S1 Text.** Complete heatmap of lncRNA target genes in REACTOME pathways which have more than 50 genes. **Figure K in S1 Text.** Expression correlations between regulatory lncRNA-mRNA pairs (S18 Table), calculated using STAR counts (y axis) or HTSeq-counts (x-axis).
(DOC)

S2 Text Null distribution of R2 values when mRNA expression was predicted using non-regulatory enzyme expression. (DOC)

S3 Text Examples of trans lncRNA-mRNA pairs that have literature support for their regulatory relationship. (DOC)

S4 Text Empirical p-value calculation for ceRNA lncRNA regulators. (DOC)
