# Supplementary material for: Quantitative estimates of the regulatory influence of long non-coding RNAs on global gene expression variation using TCGA breast cancer transcriptomic data
Source: PLoS Comput Biol. 2024 Jun 5;20(6):e1012103. doi: 10.1371/journal.pcbi.1012103 (PMC11198904; doi:10.1371/journal.pcbi.1012103)
Supplement: S1 Text — Histogram of R2 values when mRNA expression was modeled using lncRNAs only. Figure B in S1 Text. Histogram of Pearson Correlation Coefficient between real and model-predicted expression values on test samples. Figure C in S1 Text. Density scatter plot of Pearson Correlation Coefficients between predicted and real expression values. Figure D in S1 Text. Histogram of overall R2 values when residuals from TF-based models were predicted using lncRNA. Figure E in S1 Text. Histogram of test R2 values modeling permuted expression. Figure F in S1 Text. Histogram of test R2 using overlapping lncRNAs only. Figure G in S1 Text. Scatter plot of test R2 values of models using all lncRNAs and those using overlapping lncRNAs only. Figure H in S1 Text. Comparison of negative targets and positive targets. Figure I in S1 Text. Comparison of cis targets and trans targets. Figure J in S1 Text. Complete heatmap of lncRNA target genes in REACTOME pathways which have more than 50 genes. Figure K in S1 Text. Expression correlations between regulatory lncRNA-mRNA pairs (S18 Table), calculated using STAR counts (y axis) or HTSeq-counts (x-axis). (DOC) [file pcbi.1012103.s023.doc]

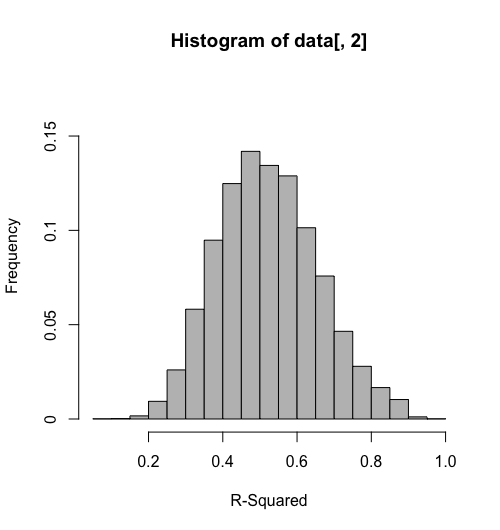


**Figure A.** Histogram of
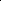
 values when mRNA expression was modeled using lncRNAs only. For each mRNA, Elastic Net was used to select ~10 lncRNAs as predictors, and the mRNA’s expression was then modeled as a linear function of expression levels of selected lncRNAs.

**Figure B.** Histogram of Pearson Correlation Coefficient between real and model-predicted expression values on test samples. Models were trained to use a selection of ~10 lncRNAs as predictors for each mRNA. Correlation coefficient was calculated five times, using 5-fold cross-validation, and the average of these five values is shown.


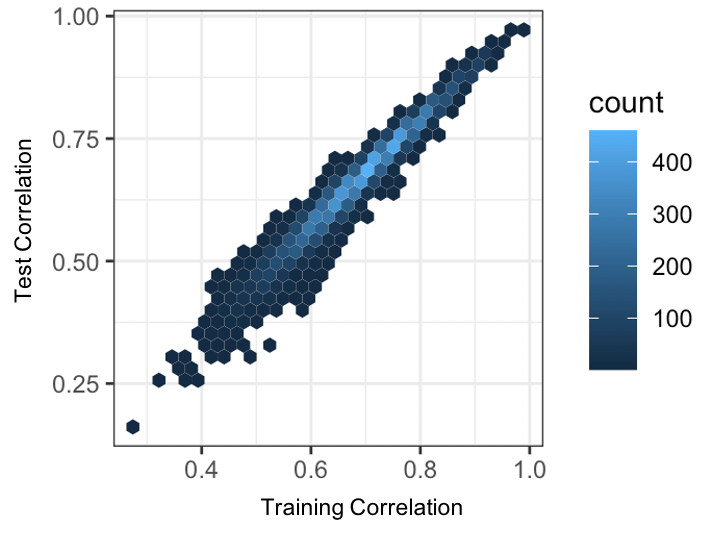


**Figure C.** Density scatter plot of Pearson Correlation Coefficients between predicted and real expression values, calculated on entire data set (x-axis) or on test samples only (y-axis). The test correlations shown are means from five folds of cross-validation.


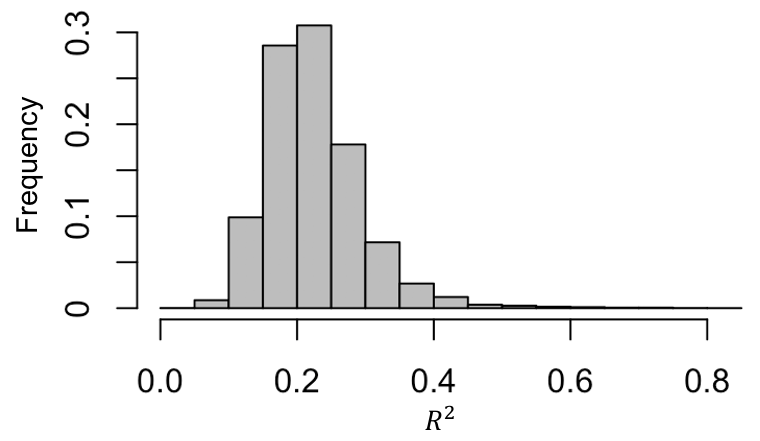


**Figure D.** Histogram of overall
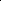
 values when residuals from TF-based models were predicted using lncRNAs, with the models being trained and then evaluated on all samples.


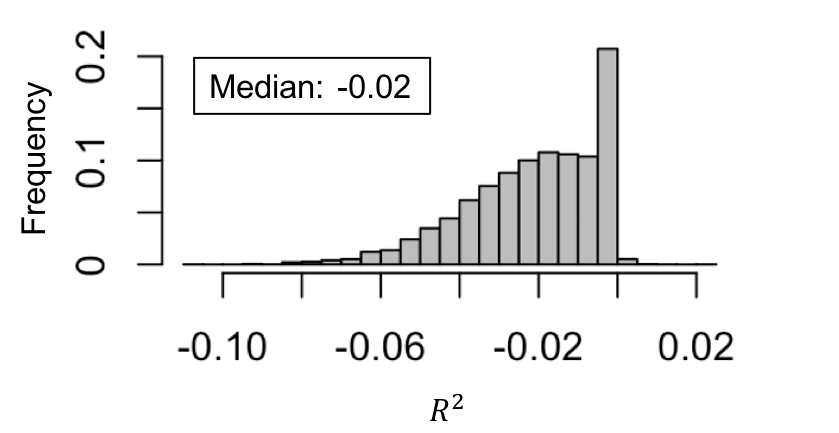


**Figure E.** Histogram of test
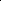
 values modeling permuted expression. Each mRNA’s expression profile (levels in different samples) was permuted prior to modeling it using lncRNAs. The modeling was performed exactly as for original profiles – lncRNAs were selected using Elastic Net and a linear model was fit to training data and evaluated on test data in a five-fold cross-validation scheme. Reported
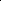
 values are averages of test
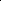
 over the five folds. The lncRNA expression used as covariates in the models was unchanged from the original data (no permutation performed). Note that
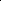
 values *can* be negative when a model is used to explain unseen data.

**Figure F.** Histogram of test
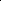
 using overlapping lncRNAs only, limited to those target genes for which using all lncRNAs achieved test
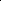
 values greater than 0.2.


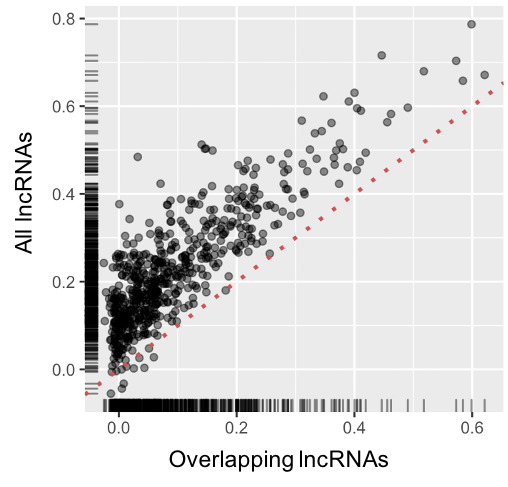


**Figure G.** Scatter plot of test
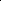
 values of models using all lncRNAs and those using overlapping lncRNAs only, limited to those target genes that had at least one overlapping lncRNA.

**Figure H**. Each point represents a lncRNA, X axis indicates the number of predicted target genes positively correlated with it and Y axis is the number of negatively correlated targets. Shown are all lncRNAs in the multi-evidence regulatory network that have at least 5 targets, i.e., X+Y >= 5. The four labeled points represent lncRNAs for which there is a significant bias towards positive or negative targets (Binomial test, nominal p-value < 0.01).


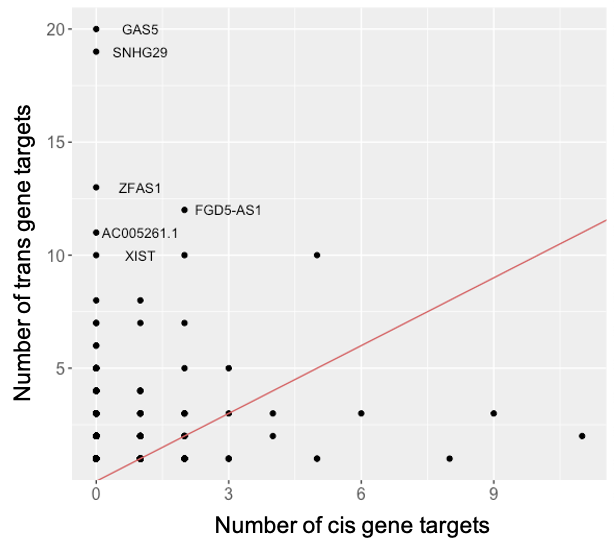


**Figure I.** The out degree of a long non-coding RNA (lncRNA) in the regulatory network represents the number of connections between the lncRNA and its target messenger RNAs (mRNAs). In this analysis, we compared the degree of connections for cis genes with the degree with trans connections. We identified several lncRNAs that exhibited notable higher trans degree. The lncRNAs with the largest disparities between the two are GAS5, SNHG29, XIST, etc.


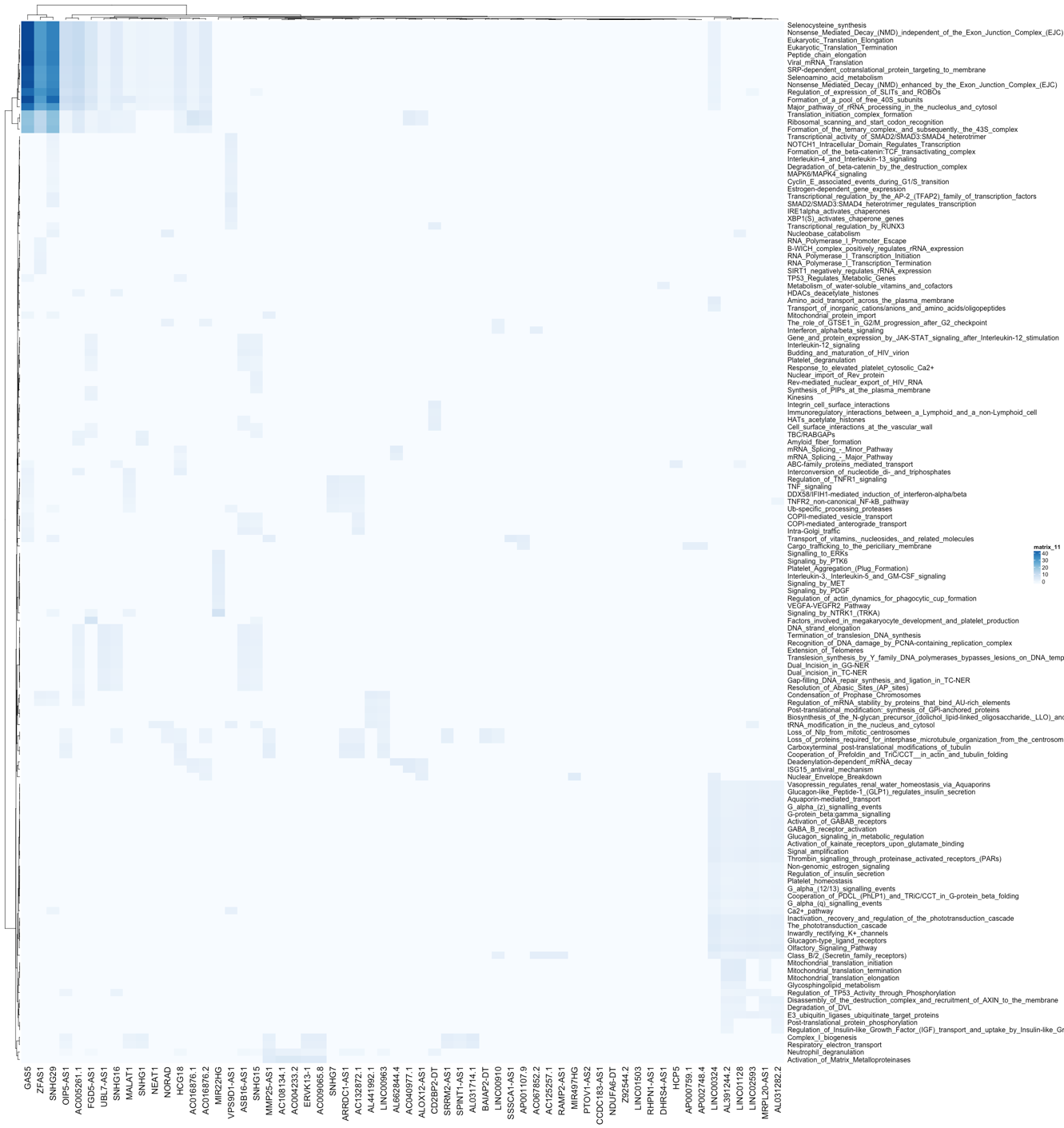


**Figure J.** Complete heatmap of lncRNA target genes in REACTOME pathways which have more than 50 genes.


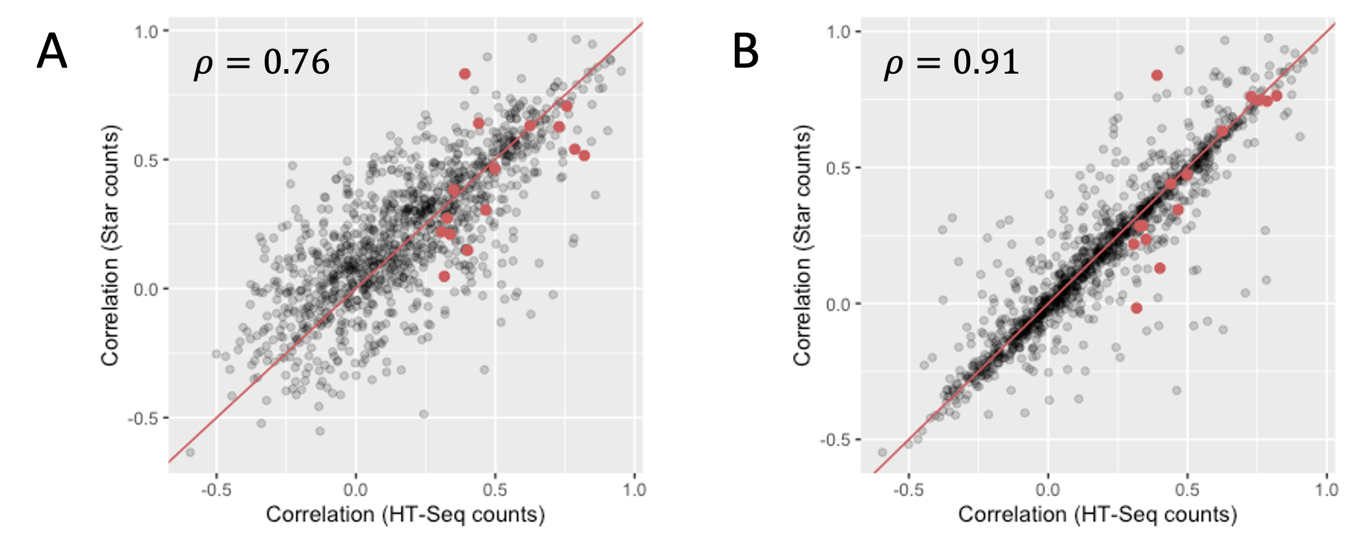


**Figure K.** Expression correlations between lncRNA-mRNA pairs reported as regulatory pairs in our work (Supplementary table S18), calculated using STAR counts (y axis) or HT-seq counts (x-axis). Red points indicate overlapping pairs. Panels A is based on STAR counts of 1st strand and panel B is based on STAR counts of 2nd strand.
