# Supplementary material for: Quantitative estimates of the regulatory influence of long non-coding RNAs on global gene expression variation using TCGA breast cancer transcriptomic data
Source: PLoS Comput Biol. 2024 Jun 5;20(6):e1012103. doi: 10.1371/journal.pcbi.1012103 (PMC11198904; doi:10.1371/journal.pcbi.1012103)
Supplement: S2 Text — (DOC) [file pcbi.1012103.s024.doc]

**S2 Text.**

We revisited the control test where each target mRNA was modeled using 1,079 enzyme-encoding, presumably non-regulatory RNAs, a null distribution of R^2^ values was obtained based on 100 repeats of such modeling, and an empirical p-value was assigned to the R^2^ value observed when modeling the same gene using lncRNAs (Supplementary Table S3). We extracted significant regulators found in these control tests, using the same criteria as for lncRNA regulators reported in Supplementary Table S4 (average test R2 >= 0.2, OLS p-value of predictor <= 1E-10, correlation absolute value >= 0.1). Across the 100 repeats of this control exercise, we observed 182.5 regulator-target associations on average, of which 1.4%, 12.2%, 25.4%, and 49.9% belonged to the “overlapping”, “same-TAD”, “proximal” and “same-chromosome” categories respectively, vis-a-vis the respective target mRNA (compare these to values in **Table 1**).
