# Supplementary material for: Quantitative estimates of the regulatory influence of long non-coding RNAs on global gene expression variation using TCGA breast cancer transcriptomic data
Source: PLoS Comput Biol. 2024 Jun 5;20(6):e1012103. doi: 10.1371/journal.pcbi.1012103 (PMC11198904; doi:10.1371/journal.pcbi.1012103)
Supplement: S3 Text — (DOC) [file pcbi.1012103.s025.doc]

**S3 Text.**

We examined the 288 lncRNA-mRNA pairs (of the 643 in Supplementary Table S4) whose genes were located more than 1 Mbp away on the same chromosome or on different chromosomes. Among these, the lncRNA with the most associations was AC010168.2, which is located within 100 bp of the histone gene HIST4H4 and has strong positive correlation with five trans located histone genes HIST1H4I, HIST2H2BE, HIST1H2BC, HIST1H4H, and HIST1H2AC (Supplementary Table S4), in addition to the proximal genes HIST4H4 and H2AFJ. (These were the only associations noted for the lncRNA.) Two other examples of strong “trans” associations included the lncRNA-mRNA pairs GAS5-RPL18A and HCP5-HLA-F. Association between the lncRNA GAS5 and the ribosomal protein RPL18A has been previously noted in low grade gliomas [1]; moreover, GAS5 has been reported to promote apoptosis and inhibit proliferation in triple negative breast cancer [2,3]. The lncRNA HCP is an HLA class I endogenous retroviral gene and HLA-F has been reported to be functionally related to it with high confidence [4], thus supporting the relationship identified above.

1. Wang Y, Xin S, Zhang K, Shi R, Bao X. Low GAS5 Levels as a Predictor of Poor Survival in Patients with Lower-Grade Gliomas. J Oncol. 2019;2019:1785042. Epub 2019/03/12. doi: 10.1155/2019/1785042. PubMed PMID: 30853980; PubMed Central PMCID: PMCPMC6377997.

2. Zheng S, Li M, Miao K, Xu H. lncRNA GAS5-promoted apoptosis in triple-negative breast cancer by targeting miR-378a-5p/SUFU signaling. J Cell Biochem. 2020;121(3):2225-35. Epub 2019/11/07. doi: 10.1002/jcb.29445. PubMed PMID: 31692053.

3. Li J, Li L, Yuan H, Huang XW, Xiang T, Dai S. Up-regulated lncRNA GAS5 promotes chemosensitivity and apoptosis of triple-negative breast cancer cells. Cell Cycle. 2019;18(16):1965-75. Epub 2019/07/10. doi: 10.1080/15384101.2019.1635870. PubMed PMID: 31282278; PubMed Central PMCID: PMCPMC6681776.

4. Kulski JK. Long Noncoding RNA HCP5, a Hybrid HLA Class I Endogenous Retroviral Gene: Structure, Expression, and Disease Associations. Cells. 2019;8(5). Epub 2019/05/30. doi: 10.3390/cells8050480. PubMed PMID: 31137555; PubMed Central PMCID: PMCPMC6562477.
