# Supplementary material for: Quantitative estimates of the regulatory influence of long non-coding RNAs on global gene expression variation using TCGA breast cancer transcriptomic data
Source: PLoS Comput Biol. 2024 Jun 5;20(6):e1012103. doi: 10.1371/journal.pcbi.1012103 (PMC11198904; doi:10.1371/journal.pcbi.1012103)
Supplement: S4 Text — (DOC) [file pcbi.1012103.s026.doc]

**S4 Text**

We first modeled each target gene using lncRNAs that are putatively associated with that gene via the ceRNA mechanism, noting down the R^2^ value achieved. The putative ceRNAs were obtained from miRNA target database TarBase v8 (see Method). We then repeated the modeling of each gene using randomly selected lncRNA candidates rather than ceRNAs. We chose as many random lncRNAs as there were candidate ceRNAs for that gene, computed the R^2^ for the model trained with these random lncRNAs as covariates, and repeated the process 100 times to obtain an empirical null distribution for the gene. This provided us an empirical p-value for the R^2^ value achieved when using its putative ceRNAs as predictors.
